# Comparative Secretome Analyses of Human and Zoonotic *Staphylococcus aureus* Isolates CC8, CC22, and CC398*[Fn FN2]

**DOI:** 10.1074/mcp.RA118.001036

**Published:** 2018-09-10

**Authors:** Tobias Busche, Mélanie Hillion, Vu Van Loi, David Berg, Birgit Walther, Torsten Semmler, Birgit Strommenger, Wolfgang Witte, Christiane Cuny, Alexander Mellmann, Mark A. Holmes, Jörn Kalinowski, Lorenz Adrian, Jörg Bernhardt, Haike Antelmann

**Affiliations:** From the ‡Institute for Biology-Microbiology, Freie Universität Berlin, D-14195 Berlin, Germany;; §Center for Biotechnology, Bielefeld University, D-33594 Bielefeld, Germany;; ¶Robert Koch Institute, Advanced Light and Electron Microscopy, D-13353 Berlin, Germany;; ‖Institute of Microbiology and Epizootics, Centre for Infection Medicine, Freie Universität Berlin, D-14153 Berlin, Germany;; **Robert Koch Institute, Wernigerode Branch, D-38855 Wernigerode, Germany;; ‡‡Institute of Hygiene, University Hospital Münster, D-48149 Münster, Germany;; §§Department of Veterinary Medicine, University of Cambridge, Cambridge CB3 0ES, UK;; ¶¶Department Isotope Biogeochemistry, Helmholtz Centre for Environmental Research-UFZ, D-04318 Leipzig, Germany;; ‖‖Chair of Geobiotechnology, Technische Universität Berlin, D-13355 Berlin, Germany;; ‡‡‡Institute for Microbiology, University of Greifswald, D-17489 Greifswald, Germany

**Keywords:** Microbiology, Pathogens, Proteogenomics, Secretome, Virulence, clonal complexes, MRSA, Staphylococcus aureus, virulence factor secretion

## Abstract

The spread of methicillin-resistant *Staphylococcus aureus* (MRSA) in the community, hospitals and in livestock is mediated by highly diverse virulence factors that include secreted toxins, superantigens, enzymes and surface-associated adhesins allowing host adaptation and colonization. Here, we combined proteogenomics, secretome and phenotype analyses to compare the secreted virulence factors in selected *S. aureus* isolates of the dominant human- and livestock-associated genetic lineages CC8, CC22, and CC398. The proteogenomic comparison revealed 2181 core genes and 1306 accessory genes in 18 *S. aureus* isolates reflecting the high genome diversity. Using secretome analysis, we identified 869 secreted proteins with 538 commons in eight isolates of CC8, CC22, and CC398. These include 64 predicted extracellular and 37 cell surface proteins that account for 82.4% of total secretome abundance. Among the top 10 most abundantly secreted virulence factors are the major autolysins (Atl, IsaA, Sle1, SAUPAN006375000), lipases and lipoteichoic acid hydrolases (Lip, Geh, LtaS), cytolytic toxins (Hla, Hlb, PSMβ1) and proteases (SspB). The CC398 isolates showed lower secretion of cell wall proteins, but higher secretion of α- and β-hemolysins (Hla, Hlb) which correlated with an increased Agr activity and strong hemolysis. CC398 strains were further characterized by lower biofilm formation and staphyloxanthin levels because of decreased SigB activity. Overall, comparative secretome analyses revealed CC8- or CC22-specific enterotoxin and Spl protease secretion as well as Agr- and SigB-controlled differences in exotoxin and surface protein secretion between human-specific and zoonotic lineages of *S. aureus*.

## Abstract (precis)

The proteogenomes and secretomes of dominant human and zoonotic *S. aureus* lineages CC8, CC22 and CC398 were compared revealing genomic and regulatory differences in the secretion of 869 proteins. In the core secretome, 101 secreted or cell surface anchored virulence factors contribute with 82.4% to total secretome abundance. CC398 isolates showed higher secretion of α- and ß-hemolysins and lower secretion of surface proteins resulting in strong hemolysis and decreased biofilm formation because of lower SigB activity compared to human-specific CC8 and CC22.

## Graphical Abstract

**Figure:**
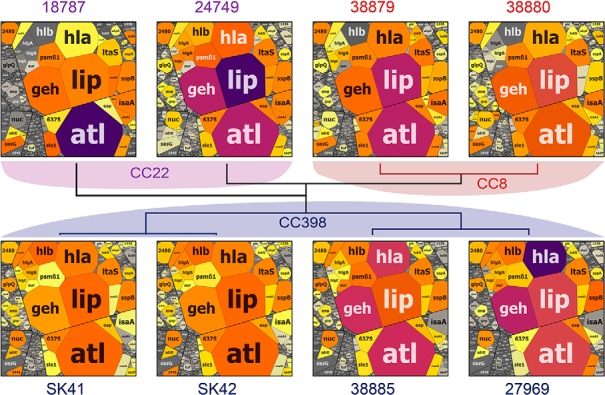

**Highlights**

- Proteogenomics and secretome comparison of human and zoonotic *Staphylococcus aureus* lineages.
- 869 secreted proteins identified in eight *S. aureus* isolates of CC8, CC22 and CC398.
- CC398 lower secretion of surface proteins and higher secretion of hemolysins and exoenzymes.
- Regulatory differences in the secretomes could be linked to lower SigB activity in CC398.

*S. aureus* is an opportunistic human and animal pathogen that colonizes the anterior nares of about 20–30% of the healthy human population (1, 2). However, *S. aureus* also causes many severe diseases, ranging from local skin and soft tissue infections to life-threatening systemic infections, such as bacteriemia, septicemia, endocarditis, osteomyelitis and necrotizing pneumonia (3–5). *S. aureus* infections are a major health burden and in case of methicillin-resistant *S. aureus* (MRSA)^1^ treatment options are limited. MRSA strains have frequently been detected in humans and livestock (6). Human MRSA strains include hospital-acquired (HA) and community-acquired (CA) MRSA isolates with increased virulence, with respect to severe soft tissue infections, such as strain USA300 (7). Moreover, patients were also found colonized and infected by epidemic livestock-associated (LA)-MRSA CC398 isolates which have emerged especially in regions with dense pig production (8–12).

The successful adaptation of *S. aureus* to various host niches requires changes in the composition of virulence factors which are often encoded on mobile genetic elements (MGE), such as prophages, plasmids and pathogenicity islands (13). MGE account for 15–20% of the *S. aureus* accessory genome and can spread rapidly between *S. aureus* isolates because of horizontal gene transfer (HGT) (14). Because the population structure of *S. aureus* is mainly clonal, multilocus sequence typing (MLST) and subsequent discrimination of sequence types and clonal complexes (CC) allows us to understand the evolutionary dynamics with respect to antibiotic resistance and virulence factor development as well as adaptation to various host niches (15, 16). CC8 and CC22 are human-dominant lineages frequently associated with community-acquired (CC8) and hospital-acquired infections (CC22) but can also be transferred to animals (17, 18). CC398 originated in humans and later adapted to livestock, where it acquired methicillin resistance, resulting in LA-MRSA (19).

Comparative genomics revealed that *S. aureus* produces highly diverse secreted and surface-associated virulence factors. The secreted virulence factors include extracellular enzymes, such as coagulase, proteases, nucleases, lipases, β-hemolysin, staphylokinase, and exfoliative toxins. Furthermore, *S. aureus* secretes many pore-forming toxins, such as α- and γ-hemolysins, Panton Valentine leukocidin (PVL), phenol soluble modulins (PSMs, δ-hemolysin), and superantigens (toxic shock syndrome toxin (TSST), enterotoxins (SE)) (20–22). Surface-associated virulence factors include covalently anchored cell wall proteins classified as “microbial surface component recognizing adhesive matrix molecules” (MSCRAMMs) (22–24) as well as non-covalently linked surface proteins with specific cell-wall binding domains, termed as “secretable expanded repertoire adhesive molecules” (SERAMs) (25). MSCRAMMs are covalently anchored to the peptidoglycan *via* sortase-based mechanisms, contain IgG-fold domains, and promote adhesion to host matrix proteins, such as fibrinogen, fibronectin and collagen (23, 24). Typical MSCRAMMs are clumping factors (ClfA/B), serine-aspartate repeat-containing proteins (SdrC/D/E), fibrinogen-binding proteins (FnBPA/B) and collagen adhesin (Cna) (23). Examples for non-covalently linked SERAMs are the extracellular fibrinogen-binding, adhesive and matrix proteins (Efb, Eap, Emp), the coagulase (Coa), cell wall hydolases and autolysins (Atl, Sle-1, IsaA), secretory antigen (SsaA) and secreted toxins (LukG, LukH), which contain various cell wall binding domains (LysM, GW, SH3B and G5) (25, 26). SERAMs are implicated in endovascular disease and elicit human antibody responses in patients with genetic blistering disease epidermolysis bullosa (25, 26). Collectively, secreted and cell wall-bound virulence factors of *S. aureus* contribute to the pathogenicity, colonization, immune evasion and resistance to host defenses (22, 27).

In previous studies, it was postulated that CC398 strains are less virulent because they do not possess enterotoxins and phage-encoded PVL, which are important virulence factors in human lineages (8, 28). Most virulence genes of CC398 strains were common among all analyzed CC's and represented the core virulence genes of *S. aureus* (28). It has been suggested that the ability of CC398 for enhanced animal colonization might be mediated by new virulence determinants (28). The comparison of porcine and human isolates of LA-MRSA CC398 with HA- and CA-MRSA strains revealed a high cytotoxic potential for CC398 strains and a high transcriptional level of *hla* and *hlb* (29). However, colonization and adherence to human cells, in particulary keratinocytes, were reduced in LA-MRSA CC398 strains compared with human MRSA (29). The secretion of intact β-hemolysin was found in the majority of livestock-associated CC398 isolates because of an intact *hlb* gene (12, 29, 30). In contrast, most human *S. aureus* clones carry the β-hemolysin-converting prophage ΦSa3, which integrates into the *hlb* locus resulting in non-functional truncated β-hemolysin variants (31). The phage ΦSa3 encodes the immune evasion cluster (IEC), including chemotaxis inhibitory protein (CHIPS), staphylococcal complement inhibitor (SCIN) and the staphylokinase (SAK) which seem to be required for human adaptation (32–36). The intact β-hemolysin in LA-MRSA CC398 isolates of livestock origin was previously found as one difference in the virulence factor equipment compared with most human clones (12, 29).

Comparative transcriptomics has been previously used to study *S. aureus* virulence factor expression in different genetic lineages. However, these results may not reflect the actual composition of secreted and cell surface-associated virulence factors, such as toxins, superantigens, extracellular enzymes and adhesins required for spreading of the pathogen and immune evasion (37). Thus, extracellular proteome (secretome) analysis is the method of choice for identification of *S. aureus* secreted and surface-attached virulence factors. Previous secretome analyses of 25 *S. aureus* patient isolates revealed a remarkably high exoproteome heterogeneity of 63 secreted proteins that is not related to genome heterogeneity (38, 39). Instead, major differences in the composition of the *S. aureus* secretome were caused by differences in transcriptional regulation by SarA- and AgrA-controlled gene expression of virulence factors, such as the hemolytic α-toxin (38, 39).

The AgrAC two component quorum-sensing system regulates expression of the RNAIII which is responsible for post-transcriptional regulation of toxins and surface proteins (40–46). Upon entry into the stationary phase, the AgrAC system is activated by autoinducer peptides resulting in RNAIII transcription. RNAIII represses genes for cell surface proteins (*e.g. spa, fnbA*) and activates toxin and protease genes (*e.g. hla, hlb, psms, sspB*) (45, 46). AgrAC was shown to promote toxin expression during acute, systemic infections and represses biofilm formation, which is associated with chronic *S. aureus* infections (40–46). The *agr* locus is highly variable and non-hemolytic *agr*-defective clinical isolates frequently arise during *S. aureus* infections indicating that biofilm formation is favored during chronic infections (47, 48). In addition, the alternative stress and stationary phase sigma factor SigmaB (SigB) has been shown to inhibit Agr activity and to promote biofilm and structured macrocolony formation (40, 42, 43).

Although the roles of Agr and SigB have been widely studied in *S. aureus* clinical isolates, the regulatory changes in virulence factor secretion between epidemic human and zoonotic dominant lineages CC8, CC22 and CC398 have not been studied in detail. The composition of virulence factors in different genetic lineages may elucidate new factors allowing adaptation of *S. aureus* to various host niches. Thus, we selected methicillin-resistant and methicillin-sensitive *S. aureus* (MRSA and MSSA) isolates of the dominant human and livestock genetic lineages CC8, CC22 and CC398 from various infection sites for comparative secretome analysis using label-free quantitative mass spectrometry. The secretome comparison revealed CC-specific virulence factors that are specific for dominant human- or livestock-associated lineages of CC8, CC22 and CC398, but also expression differences because of different Agr and SigB activities. We further investigated the virulence phenotypes of the *S. aureus* isolates and revealed higher hemolysis, lower biofilm formation and less pigmented macrocolony phenotypes in most CC398 isolates that could be linked to different SigB and Agr activities in support of our secretome data.

## EXPERIMENTAL PROCEDURES

### 

#### 

##### Bacterial Strains and Growth Conditions

The 18 *S. aureus* isolates of CC398, CC8 and CC22 were obtained from humans, horse and pigs from wound, invasive and bacteraemic infections. The strain collection was received from the Robert Koch Institut (RKI) (Wernigerode branch) and from the Institute of Microbiology and Epizootics Berlin (IMT) (Table I). All isolates were previously analyzed by multi-locus sequence typing (MLST) for their *spa*-types and for the presence of *mecA*, phage-encoded immune evasion cluster (IEC) genes (*sak, scn, chp*) and PVL genes (*lukF-PV* and *lukS-PV*) (Table I). The *S. aureus* isolates used are five human strains of CC8 from invasive infections of *spa* t008 (IMT numbers 38876, 38877, 38878, 38879, 38880) and five human isolates of CC22 from the nose, invasive and wounds infections of *spa* types t005, t032 and t310 (18787, 24749, 27969, 38881, 38882, 38883). We further used human, horse and pig CC398 isolates from the nose, septicemia, pneumoniae, sinusitis and abscesses of *spa* types t011, t6867, t12359 (27969, 38884, 38885, 38886, 38887, 38888, 38951-SK41, 40439-SK42) (Table I). The two pig isolates SK41/SK42 represent an isogenic MRSA/MSSA pair with the MRSA strain SK41 and the secondary MSSA strain SK42 that has lost the SCC*mec* element during subcultivation. For comparison of virulence phenotype assays, we used the CA-MRSA strain USA300 (49) and *S. aureus* 8325–4 which is cured of prophages and has an 11 bp deletion of *rsbU* (50). The *S. aureus* isolates were cultivated in TSB medium for the analysis of the extracellular proteome, biofilm and macrocolony formation, hemolytic activity and staphyloxanthin levels as described below. Sheep blood and crystal violet were obtained from Oxoid and Roth, respectively.

##### Genome Sequencing of the CC398 S. aureus Isolates

The CC398 isolates were whole-genome sequenced (WGS) using Illumina MiSeq 300 bp paired-end sequencing with an obtained coverage > 90X. After quality control using the NGS tool kit (51) (70% of bases with a phred score >20), high-quality filtered reads were used for *de novo* assembly into contiguous sequences (contigs) and subsequently into scaffolds using SPAdes v3.9 (52). Assembled draft genomes of the isolates were annotated using Prodigal (PROkaryotic DYnamic programming Gene-finding ALgorithm) (53). The Whole Genome Shotgun project of the genome's sequences of the eight CC398 isolates 27969, 38884, 38885, 38886, 38887, 38888, 38951-SK41, 40439-SK42 has been deposited at DDBJ/ENA/GenBank under the accession numbers QBFK00000000, QBFL00000000, QBFM00000000, QBFN00000000, QBFKO0000000, QBFP00000000, QBFQ00000000, QBFR00000000. The sequence data of the five CC8 and five CC22 isolates have been previously published and are available in the European Nucleotide Archive (ENA) (http://www.ebi.ac.uk/ena/) under accession numbers ERP000150, ERP000633, PRJEB14816 (54–56).

##### Sample Preparation for the Extracellular Proteome Analysis By LC-MS/MS

The *S. aureus* isolates were grown in TSB medium and the cells were harvested during the stationary phase after 9 h of growth by centrifugation at 10000 g for 10 min at 4 °C. The cell-free supernatants with the extracellular proteins were filtered using a 0.22 μm micropore filter. The extracellular proteins were precipitated using 10% trichloroacetic acid (TCA) overnight at 4 °C and harvested by centrifugation at 4 °C for 30 min at 13000 g. The TCA-precipitated exoproteins were washed three times in ice-cold 70% ethanol. The exoproteins were dissolved in a solution containing 7 m urea, 2 m thiourea and 100 mm dithiothreitol. Samples of 20 μg proteins were separated using a short 15% SDS-PAGE run for 15 min and the gels were stained with Coomassie Blue. The gel lanes with the exoproteins were cut into three fractions and used for in-gel tryptic digestion as described previously (57). The tryptic peptides were extracted using a solution containing 0.5% (w/v) trifluoroacetic acid and 50% (v/v) acetonitrile as described (57). The eluted peptides were desalted using ZipTip-μC18 material (Merck Millipore, Darmstadt, Germany) and resuspended in 0.1% (v/v) formic acid before LC-MS/MS analysis. The peptide solution was subjected to nLC-MS/MS analysis using an Orbitrap Fusion (Thermo Fisher Scientific, Waltham, MA, USA) coupled to a TriVersa NanoMate (Advion, Ltd., Harlow, UK) as described previously (58). In total, 5 μl of the peptide solution were separated with a constant flow of 300 nL/min on a 15 cm analytical column (Acclaim PepMap 100 RSLC, 2 μm C18 particles, nanoViper connections, 75 μm x 25 cm Thermo Scientific) at 35 °C using a Dionex Ultimate 3000 nano-LC system (Dionex/Thermo Fisher Scientific, Idstein, Germany). The gradient was made of solvent A (0.1% formic acid) and solvent B (80% acetonitrile, 0.08% formic acid) in a linear 90 min gradient of 4% to 55% solvent B.

##### Experimental Design and Statistical Rationale

In this study, we compared the extracellular proteome fractions of six human and two pig *S. aureus* isolates of CC8, CC22 and CC398. The extracellular proteomes of these eight *S. aureus* isolates were analyzed from 3 biological replicate experiments for each strain, resulting in 24 total exoproteome datasets. The 24 exoproteome samples were harvested during the stationary phase after 9 h of growth and analyzed using mass spectrometry-based label-free quantification (LFQ). Each of these 24 extracellular proteome samples was present in 3 analytical fractions that derived from 3 gel-slices cut from 1D-gels and used for in gel-digestion as explained above. The 3 analytical peptide fractions per exoproteome sample were combined before the LC-MS/MS run and measured together as one sample for the same isolate. All MS raw files were analyzed against an in-house pan-proteogenome FASTA-file (see next section) using the Andromeda search engine integrated into the MaxQuant software. LFQ intensity values were quantified for 3635 proteins in 3 biological replicates for the eight isolates and used for calculation of average values and standard deviations for each isolate (supplemental Table S6). We also considered 647 proteins quantified in 2 biological replicates for calculation of average LFQ intensity values when the proteins were quantified in other isolates in 3 biological replicates. Proteins identified with unique peptides in at least 2 out of 3 replicates were manually approved for their MS/MS spectra using MS Viewer (supplemental Fig. S1). For the secretome comparison, the LFQ intensities were further normalized as 99 percentile values in Excel which are the sum of 99% of all protein LFQ intensity values in each isolate to avoid bias of highly abundant proteins in one isolate (supplemental Table S8). Differentially abundant proteins in the secretomes of CC8, CC22 and CC398 were determined using a log2 fold-change cut-off threshold of >+1.5 and <−1.5 (*p* value <0.05) using the GraphPrism software. Statistical significance was determined by multiple t-tests using the Holm-Sidak method with alpha = 0.05.

##### Generation of the S. aureus Proteome Database and Label-free Quantitative Extracellular Proteome Analysis Using MaxQuant Searches

The annotated coding sequences for each of the 18 *S. aureus* genomes were used as an input to the ROARY pipeline (Rapid large-scale prokaryote pan genome analysis) (59) with a threshold for assigning unique gene identifier to CDS set at 95%. The resulting pan-proteogenome contained 4118 unique genes that were further clustered into homologues based on a threshold of 70% identity on the protein level and annotated with corresponding SAUPAN IDs extracted from the Aureowiki database (60). Those genes without a match in the SAUPAN reference collection were similarly clustered with the same threshold and assigned an artificial identifier as RKI number. This resulted in 3487 unique protein clusters that are present or absent in the 18 *S. aureus* isolates of CC8, CC22 and CC398 which are listed in supplemental Table S3, but without allele variants. The 3487 unique proteins are present in 3820 unique allele variants which are summarized in supplemental Table S4.

For the MaxQuant search, we used the complete pan-proteo-genome FASTA-file of 4118 unique SAUPAN-IDs and RKI numbers of the 18 *S. aureus* isolates obtained from the translated genomes using the ROARY analysis. The pan-proteogenome FASTA-file was concatenated with a reverse decoy database and common contaminants and used for the search of all MS raw data files using the search engine Andromeda and the MaxQuant software (version 1.5.1.2) (61–63). Quantification of LFQ intensities of the peptides in the 24 exoproteome samples was performed using label-free quantitative proteomics.

Methods for label-free relative quantification are mainly based on spectral counting or extracted ion chromatogram (XIC)-based quantification (64–70). Spectral counting relies on the number of fragment ion spectra acquired for peptides and the comparison of their quantities between different samples. The XIC-based quantification measures peak areas for peptide precursor ions, termed as area under the curve (AUC) because the AUC is linearly correlated with peptide abundances. For LFQ exoproteomics analysis we used XIC-based quantification for which high mass resolution and accuracy is a key factor for the quality of label-free quantification (65, 71).

Data pre-processing of Maxquant output data (data filtering, log2 transformation, grouping and filtering valid values) was performed with the Perseus module version 1.5.3.2. (http://www.perseus-framework.org). Standard settings were used for MaxQuant searches. Enzyme specificity was selected to trypsin. Two miscleavages were allowed, the parent ion mass tolerance was 10 ppm and the fragment ion mass tolerance was 0.5 Da. The database searches were performed with carbamidomethylations of cysteine as fixed modification and methionine oxidation as variable modification. At least 2 peptides per protein were required for positive protein hits. Peptides were considered as identified with a false discovery rate 0.01% (FDR, Percolator). Peptide identifications were further propagated across fractions using the match between runs option which allows the transfer of peptide identification to unsequenced or unidentified peptides by matching their mass and retention time following LC-MS run alignments determined by hierarchical clustering (72). The average values and standard deviations of peptide intensities were calculated from 2–3 biological replicates and the GraphPad Prism software was used for statistical analysis as described above. A complete list of all peptide and protein identifications of the *S. aureus* isolates is available in supplemental Tables S1 and S2*A*, S2*B*, S2*C*. The supplemental Table S2*A*, S2*B* includes all protein identifications obtained in the MaxQuant search that were not filtered. Supplemental Table S2*C* includes the final list of the 869 protein identifications with number of unique peptides, % sequence coverage and LFQ intensity values that are used for further analysis in supplemental Table S6. Amino acid sequence comparisons were made using the BlastP (basic local alignment search tool) algorithm (blast.ncbi.nlm.nih.gov). The subcellular localization of the secreted proteins was predicted by PSORTb version 3.0.2 (73). The MS raw files have been deposited to the ProteomeXchange Consortium via the PRIDE (74) partner repository with the dataset identifier PXD008797. The MS/MS spectra of all identified peptides have been deposited to the MS Viewer database (http://msviewer.ucsf.edu/prospector) under the key r1mmcmhlpi.

##### Voronoi Treemap Construction of the S. aureus Secretome Data

The Voronoi treemaps of the secretomes were generated using the Paver software as described (75) to visualize the 99 percentile normalized values of protein abundances for all identified extracellular proteins in each secretome of the eight isolates of CC8, CC22 and CC398. The protein abundances are shown as 99 percentile normalized values calculated in Excel which is a variant of the total count normalization approach (supplemental Table S8). For this 99-percentile normalization, all LFQ intensity values for each isolate are sorted in an increasing order and summed up beginning at the weakest and ending at the highest of 99% LFQ intensity values. All sorted LFQ intensity values within the strain specific datasets are divided now by the sum of 99% of the weakest LFQ intensity values and calculated in percentage. This 99% percentile normalization should avoid data bias because of high abundance of single proteins that are overrepresented in the secretome of specific isolates. A protein value of *e.g.* 50 indicates that this protein is 50% of the sum of 99% of lower expressed proteins. Average normalized values of protein abundances of all strains are represented as cell sizes. The color code shows the 99% percentile normalized protein abundances per strain.

##### Hemolysis Assays of S. aureus Supernatants for Hla and Hlb Activities

*S. aureus* isolates were grown in TSB medium and cells harvested by centrifugation during the stationary phase after 14 h of growth. The culture supernatants of the *S. aureus* isolates were filtered and 50 μl was added to 1 ml of 2% sheep blood in 10 mm Tris-HCl pH 7.5; 0.9% NaCl and incubated for 30 min at 37 °C. The remaining non-lysed blood cells were sedimented by centrifugation at 6000 rpm for 5 min at 4 °C. The hemolytic activity was determined by measuring the optical density at 405 nm (OD_405_) of the blood cell-free supernatant. As positive hemolysis control, a 0.1% Triton-X100 solution was used, and fresh TSB medium served as negative control in the blood cell hemolysis assays. The hemolytic activities of the *S. aureus* supernatants were normalized to the Triton-X100 positive control, which was considered as 100% hemolysis. The β-hemolysis of the *S. aureus* CC398 supernatants was also assessed in sheep-blood agar plates, because β-hemolysin is known as hot-cold toxin that interacts with sheep blood cells at 37 °C but does not lyse the cells. Further incubation at 4 °C for few hours is required to complete β-hemolysis, leading to lysis of the sheep blood cells (76, 77).

##### Biofilm Formation in Microtiter Plates and Macrocolony Formation of S. aureus Isolates

For biofilm formation using standard microtiter plates and crystal violet staining, *S. aureus* isolates were grown in TSB medium over night into the stationary phase and the OD_580_ was measured the next morning. The *S. aureus* isolates were diluted in TSB medium with 1% glucose to an OD_580_ of 0.5 and 200 μl were transferred in triplicate into a sterile flat-bottomed 96-well microtiter plates. The microtiter plates with the *S. aureus* isolates were incubated overnight at 37 °C. Biofilm formation in microtiter plates was determined by crystal violet staining as described previously (78). Briefly, after gently washing the settled biofilm cells with 0.9% NaCl solution and staining the biofilms with 0.1% crystal violet, the biofilm-forming cells were disrupted with a 1% SDS solution. The crystal violet stained lysed biofilm solution was measured at OD_595_ for quantification of biofilm formation. Biofilm formation was also investigated using structured macrocolonies on TSB agar with 100 mm MgCl_2_ as described previously (40, 41). In brief, 2 μl of cell suspension of *S. aureus* overnight cultures were spotted on TSB-MgCl_2_ agar plates and macrocolonies grown for 5 days.

##### Staphyloxanthin Extraction of S. aureus Isolates

Extraction of staphyloxanthin was performed from 1 ml of *S. aureus* overnight cultures grown in TSB medium as described (41). The cell pellet was washed and resuspended in 1 ml PBS buffer and the OD_600_ was determined. Staphyloxanthin was extracted by shaking the cell pellet 3 times in 250 μl methanol at 14,000 rpm and 55 °C for 3 min. The staphyloxanthin extract was measured at 463 nm using the CLARIOstar microplate reader (BMG Labtech). Relative staphyloxanthin levels were calculated as 463/600 nm ratios.

##### Live/Dead Viability Assay

The viability and lysis of *S. aureus* isolates during the stationary phase was analyzed using the LIVE/DEAD^®^ BacLight™ Bacterial viability kit (Thermo Fisher Science) according to the instructions of the manufacturers. In brief, *S. aureus* strains were harvested during the stationary phase after 9 h of growth, washed twice in PBS and stained with SYTO9 and propidium iodide for 15 min. Stained *S. aureus* cells were washed 3 times in PBS and live or dead cells were visualized using a fluorescence microscope (Nikon, Eclipse, T*i*2). Fluorescence intensity was measured after excitation at 488 nm (SYTO9) and 555 nm (propidium iodide) and false-colored in green or red for live or dead cells, respectively.

##### Transcriptional Analyses Using Northern Blots

Northern blotting analyses were performed as described previously (79) using RNA isolated from the *S. aureus* strains that were harvested after 3 and 6 h of growth in TSB medium. Hybridizations were performed with the digoxigenin-labeled antisense RNA probes specific for the transcripts of RNAIII, *hla, spa, asp23*, and *sigB* synthesized *in vitro* using T7 RNA polymerase as described previously (80). The primer pairs used for generation of the digoxygenin-labeled RNA probes are RNAIII-for (5′-GAAGGAGTGATTTCAATGGCAC-3′) and RNAIII-rev (5′-CTAATACGACTCACTATAGGGAGACTGAGTCCAAGGAAACTAACTC-3′), hla-for (5′-GTCAGCTCAGTAACAACAACACT-3′) and hla-rev (5′-CTAATACGACTCACTATAGGGAGACCAATTTGTTGAAGTCCAGTGC-3′), spa-for (5′-AGATCAACAAAGCGCCTTCT-3′) and spa-rev (5′-CTAATACGACTCACTATAGGGAGAACGACATGTACTCCGTTACCA-3′), asp23-for (5′-AGTAATCGTGGGGTTCCTGT-3′) and asp23-rev (5′-CTAATACGACTCACTATAGGGAGATCCGAACGAATTGCTTCAGT-3′), sigB-for (5′-AGTGTACATGTTCCGAGACGT-3′) and sigB-rev (5′-CTAATACGACTCACTATAGGGAGATTGCCGTTCTCTGAAGTCGT-3′).

## RESULTS

### 

#### 

##### Phylogenomic Comparisons of 18 S. aureus Isolates of CC8, CC22, and CC398

**Fig. 1.**
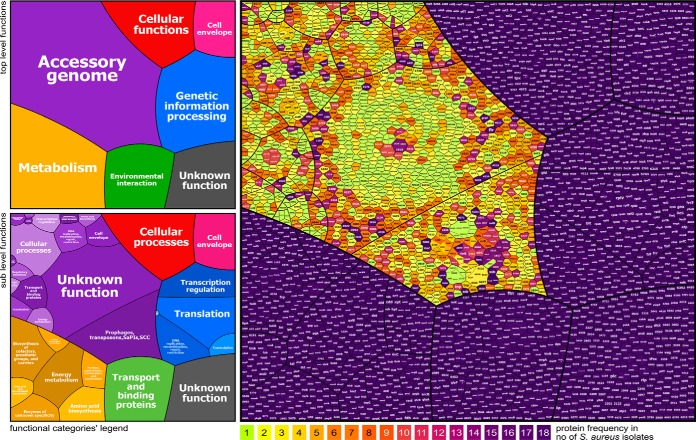
**The core and accessory proteogenomes of 18 *S. aureus* CC8, CC22, CC398 isolates.** The protein occurrence treemap (right) visualizes all 3487 unique proteins based on their presence and absence in the proteogenomes of the 18 *S. aureus* isolates. The 3487 unique proteins were sorted into functional categories based on TIGRfam and Aureowiki annotations (Table S3) (60). High diversity of the proteogenome across 18 *S. aureus* isolates is indicated in this protein occurrence treemap showing 2181 core and 1306 accessory proteins. The core proteins are colored in violet and the accessory proteins are color-coded based on their frequencies in <18 isolates. The protein frequency color code is shown below the treemap. Two legends of the treemap are presented on the left showing “top level functions” (TIGRfam level 1) and “sub level functions” (TIGRfam level 2) of the proteins as classified in Table S3. The colors in the legends (left) differentiate the functional classes of all proteins and are not related to the color code of the protein frequency treemap (right).

In this study, we aimed to compare virulence factor secretion of 18 *S. aureus* isolates frequently associated with community-acquired (CC8), hospital-acquired (CC22) and livestock infections (CC398). To gain insights into the phylogenomic differences across the dominant human and zoonotic genetic lineages CC8, CC22 and CC398, we selected 18 *S. aureus* strains from various infection sites in human, horse and pigs (Table I). First, we mapped all translated protein FASTA files encoded by the genomes of the 18 isolates to the SAUPAN-ID of the Aureowiki database (60). This pan-proteogenome FASTA database was used for comparison of the proteogenomes and for the search of the proteomic data. In addition, all proteins of the pan-proteogenomes (core and accessory proteins) were sorted into functional categories based on TIGRfam (version 15.0) and Aureowiki annotations (60). The proteogenome comparisons of the 18 *S. aureus* isolates are shown in a presence/absence Table indicating the frequency of all proteins in 18 *S. aureus* isolates (supplemental Table S3). In addition, we extracted the virulence factors, including cytolytic toxins, superantigens and enterotoxins, toxin-antitoxin systems, immune evasion cluster proteins, surface-associated proteins with MSCRAMM and SERAM functions, and other proteins annotated as virulence factors to highlight the diversity of the pathogenicity related factors in the 18 isolates (supplemental Table S5). The frequencies of all 3487 unique core and accessory proteins present or absent in the 18 isolates are displayed in Voronoi protein occurrence treemaps based on a color code (Fig. 1). This proteogenomic comparison confirmed the high genome diversity between the 18 *S. aureus* isolates of CC8, CC22 and CC398 (Fig. 1).

**Table I:**
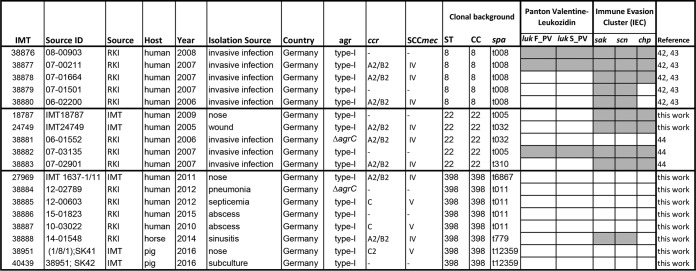
Staphylococcus aureus isolates of CC8, CC22 and CC398 used for this study

The *S. aureus* strains were obtained from the Robert Koch Institut (RKI) of Berlin and Wernigerode and of the Institute of Microbiology and Epizootics (IMT) Berlin.

The *S. aureus* isolates were analyzed by MLST and PCR for their clonal complex (CC), sequence type (ST), *spa*-type, PVL and IEC presence (grey) or absence (white).

The types of *agr*, SCC*mec* element and the *ccr* recombinases are indicated in all MRSA isolates. Ccr are required for integration and excision of the SCC*mec* element.

**Fig. 2.**
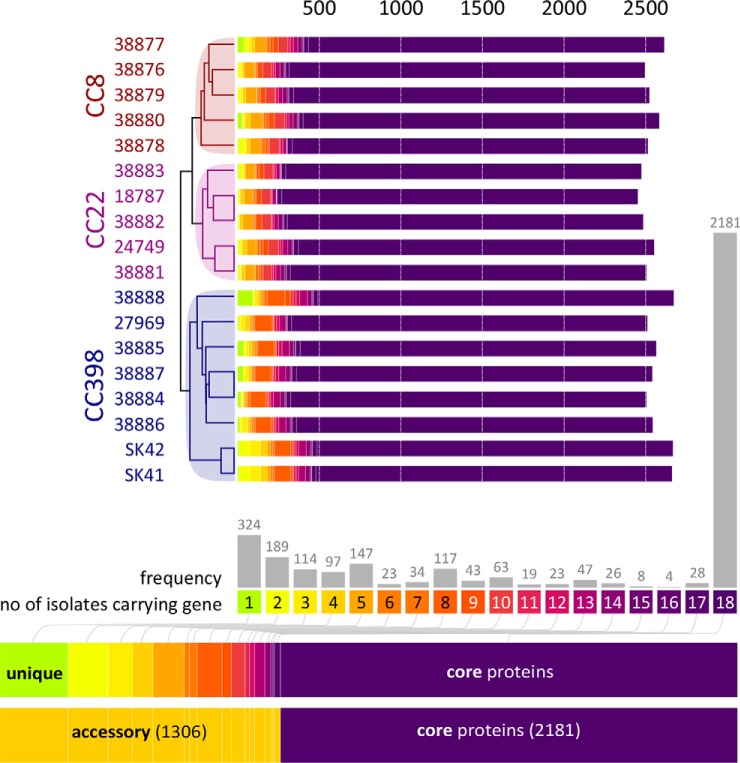
**Phylogenomic clustering of the proteogenomes of 18 *S. aureus* CC8, CC22, CC398 isolates.** Phylogenetic clustering of the proteogenomes of the 18 *S. aureus* isolates is based on protein frequencies, revealing lineage-specific branches. In total, 2181 and 1306 proteins belong to the core (violet) and accessory proteogenomes, respectively. Accessory proteins are present in <18 isolates and are color-coded based on their frequencies in the 18 isolates. The numbers of the accessory proteins that are present in 1–17 genomes are given as frequencies. The phylogenetic tree is based on the FASTA proteome data translated from the 18 genome sequences that are mapped to the PAN-IDs of the Aureowiki database. The presence and absence of all unique proteins in the 18 isolates is shown in supplemental Table S3 that was used for this phylogenomic clustering.

In total, 2181 proteins could be allocated to the core proteogenome and 1306 belong to the accessory proteogenome present in <18 isolates. The protein occurrence treemap reflects the high abundance of MGE. Phylogenetic clustering of the 3487 proteins based on their presence and absence in the *S. aureus* isolates resulted in distinct nodes that resembled the three lineages (Fig. 2). These lineage-specific branches agree with the *spa*-types of the isolates. Interestingly, the SK41 and SK42 isogenic MRSA and MSSA pair showed the highest number of accessory proteins. Of note, 627 proteins are present in only 1–3 isolates indicating the high genome diversity in different *S. aureus* lineages and isolates. About 422 proteins were newly assigned with RKI numbers because these were not present in the Aureowiki database (supplemental Table S3, S4).

##### Comparative Secretome Analysis of Eight S. aureus Isolates of CC8, CC22, and CC398 Revealed High Protein Abundances of Secreted and Surface-associated Virulence Factors in the Core Secretome

To analyze the secreted virulence factors in the secretomes, we selected two CC8 isolates (38879 and 38880), two CC22 isolates (24749 and 18787) and four CC398 isolates (27969, 38885, SK41, and SK42). The extracellular proteins of the *S. aureus* isolates were analyzed using LC-MS/MS analysis and label-free quantification (LFQ).

**Fig. 3.**
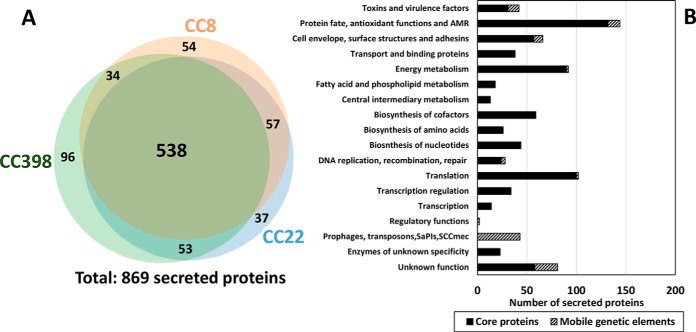
**Overview and functional categories of the 869 secreted proteins that are quantified in the secretomes of eight *S. aureus* isolates of CC8, CC22 and CC398.** *A*, The Venn diagram shows the numbers of proteins that were quantified in the secretomes of the eight *S. aureus* isolates of CC8 (38879, 38880), CC22 (17787, 24749) and CC398 (38885, 27969, SK41 and SK42) using label-free quantitative proteomics. Most identified virulence factors belong to the core genome while also unique proteins were identified that define lineage-specific secreted proteins. The Venn diagram was generated using the BioInfoRX Venn diagram plotter (http://bioinforx.com/free/bxarrays/venndiagram.php#). *B*, The 869 secreted proteins were classified according to TIGRfam and Aureowiki annotations including also core and accessory proteins (supplemental Tables S6, S8).

In total, the levels of 869 proteins could be quantified in the secretomes of the eight *S. aureus* isolates (Fig. 3*A*, supplemental Table S6). The secreted proteins were classified into functional groups based on TIGRFam and Aureowiki annotation (60) (supplemental Table S6). The majority of 538 proteins (61.9%) were found in all *S. aureus* isolates. Interestingly, there were fewer common proteins shared between CC398 and CC8 isolates compared with CC22 and CC8 or CC22 and CC398. These results correspond with the phylogenetic clustering of the proteogenomes of the 18 *S. aureus* isolates which revealed a closer phylogenetic link between CC8 and CC22 isolates (Fig. 2). Among the 869 secreted proteins are 42 toxins and other virulence factors (4.8%) (Fig. 3*B*). In total, 109 secreted proteins (12.5%) are encoded by the accessory genome. About 132 secreted proteins (16.6%) are involved in protein quality control, detoxification, antioxidant functions and antibiotic resistance. Among the cell envelope proteins and surface-associated adhesins, 57 proteins were identified that are required for pathogenicity and colonization of *S. aureus*. Overall, we identified many 607 cytoplasmic proteins (70%) that are involved in many cellular functions, such as energy metabolism, the biosynthesis of amino acids, fatty acids, nucleotides and cofactors, replication, transcription and translation (Fig. 3*B*, supplemental Table S6).

**Fig. 4.**
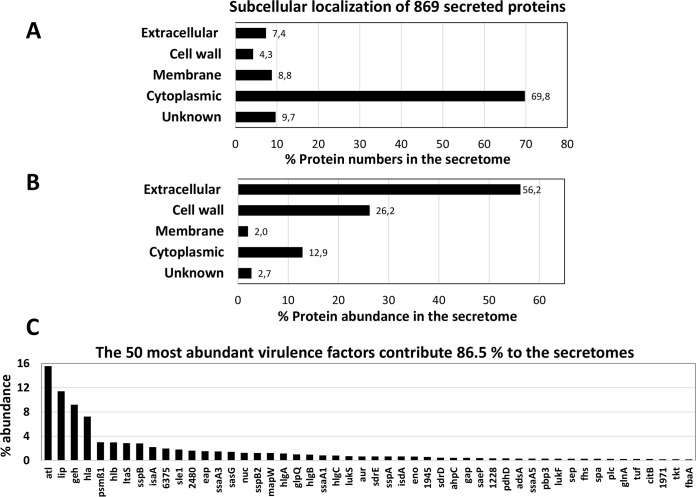
**The subcellular localization of 869 secreted proteins (*A, B*) and the 50 most abundant secreted virulence factors in all secretomes of the eight isolates of CC8, CC22 and CC398 (*C*).** *A*, *B*, The 869 secreted proteins of the eight *S. aureus* isolates were analyzed by PSORTb version 3.0.2 (73) for their subcellular localization into predicted extracellular, cell wall-associated, membrane and cytoplasmic proteins (supplemental Table S6). The diagrams show the distribution of proteins in the different compartments based on their percentages of protein numbers (*A*) and percentages of protein abundances (*B*) in relation to all proteins identified in the total secretome. This indicates that predicted 64 extracellular and 37 cell wall proteins represent with 82.4% the most abundant secreted proteins. Although 70% of proteins were identified as cytoplasmic proteins, these are low abundant and contribute only with 12.9% to the total secretome. *C*, The 50 most abundantly secreted extracellular proteins are listed according to their amounts that constitute 86.5% of the total secretome including the major autolysins (Atl, Sle1, IsaA, 6375), lipases and lipoteichoic acid hydrolase (Lip, Geh, LtaS), hemolytic toxins (Hla, Hlb, PSMβ1) among the top ten secreted virulence factors (supplemental Table S8). Thus, the majority of the secretome is highly conserved across different genetic lineages.

The subcellular localization of the identified proteins was predicted using PSORTb version 3.0.2 (73) (http://www.psort.org/psortb/) (supplemental Table S6, S7). Among the 869 identified proteins, 64 proteins (7.3%) were predicted to have an extracellular localization based on the presence of an N-terminal signal peptide and no retention signal for membrane or cell wall anchoring. In addition, 37 proteins (4.2%) were predicted as cell surface-associated, 76 proteins (8.7%) were membrane proteins and 607 (70%) were predicted as cytoplasmic proteins (Fig. 4*A*). However, the lower number of 20.2% predicted secreted, surface-associated and membrane proteins *versus* 70% cytoplasmic proteins must be normalized to their relative protein abundances in the secretome for the exact secretome composition.

Thus, we calculated the 99 percentile normalized protein abundances of all secreted proteins in the secretomes for each of the eight *S. aureus* isolates to determine which virulence factors are most abundantly secreted. The normalized protein abundances of all 869 secreted proteins of the eight *S. aureus* isolates were calculated based on their LFQ intensity values (Table II; supplemental Tables S6, S8). Next, the percentages of the protein abundance values in each secretome were calculated for the subcellular distribution of proteins. Based on these calculations, the 64 extracellular and 37 cell wall proteins contribute 56.2% and 26.2%, respectively, to the abundance of proteins in the secretomes (Fig. 4*B*). In contrast, the 607 cytoplasmic proteins contribute only with 12.9% to the secretome. Thus, the secreted and cell-surface attached virulence factors make the greatest contributions to the secretome in all isolates and can be considered as core secretome in all isolates.

**Table II:**
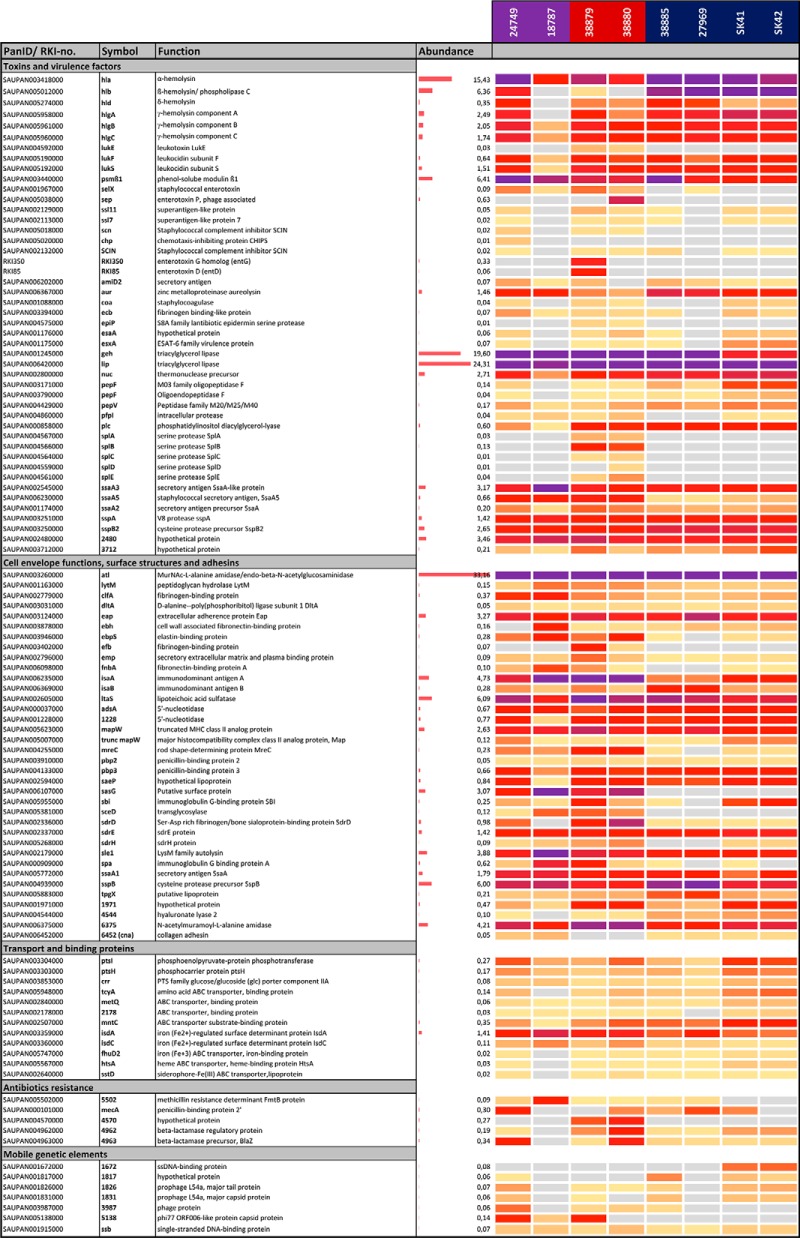

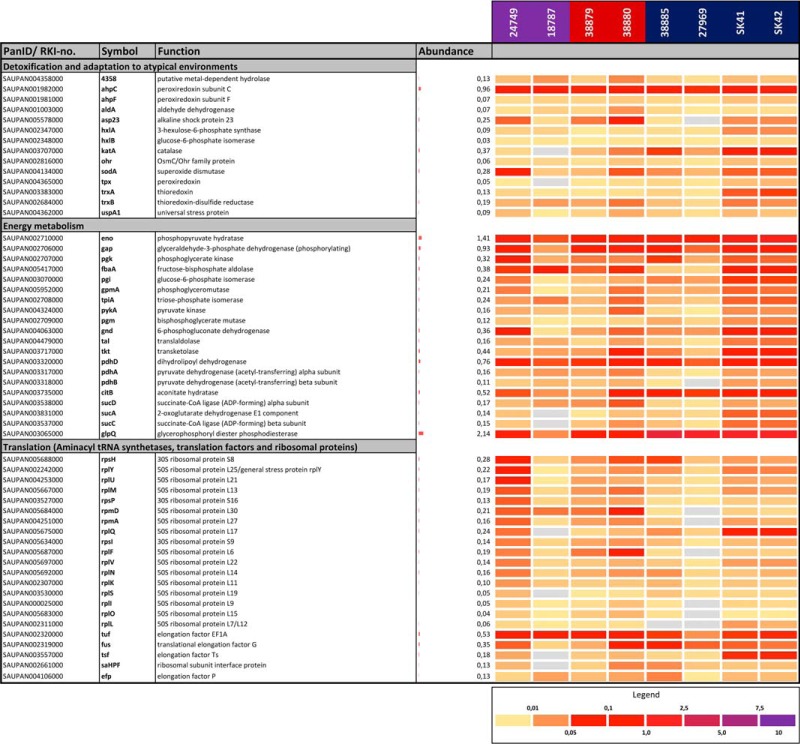
Subsets of secreted virulence factors and other proteins identified in the secretomes of the eight S. aureus isolates of CC8, CC22 and CC398

The proteins were assigned with SAUPAN-IDs and gene symbols according to the Aureowiki database. Proteins not present in the Aureowiki database were labeled with RKI numbers. The average 99 percentile protein abundances are shown for the selected secreted proteins. The full list of 869 secreted proteins identified in the secretomes and their 99 percentile protein abundances are shown in supplemental Tables S6–S8.

**Fig. 5.**
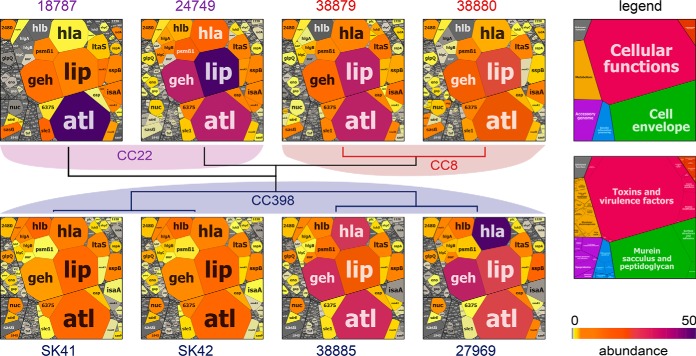
**Voronoi secretome treemaps for comparison of the secretomes of the eight *S. aureus* isolates of CC8, CC22 and CC398.** The secreted proteins that were quantified in the secretomes of the eight *S. aureus* isolates of CC8, CC22 and CC398 are visualized in Voronoi secretome treemaps. The secreted proteins were classified in functional categories according to TIGRfam and Aureowiki annotations as shown in supplemental Tables S6. The LFQ intensities of each protein were used for calculation of the normalized 99 percentile protein abundance in each of the eight secretomes (supplemental Table S8). The normalized 99 percentile of each protein across the 8 secretomes denotes the cell size of the treemap. The yellow-orange-magenta color code indicates the normalized 99 percentile abundance of the proteins in the secretomes of the different *S. aureus* isolates. The eight secretome profiles were also used for phylogenetic clustering indicating lineage-specific groups of secreted proteins. These CC8, CC22 and CC398 specific clusters of secretome profiles identified CC-specific secretome profiles because of genomic and regulatory differences. The higher secretion of toxins (Hla and Hlb) and lower secretion of many surface-associated proteins points to a higher Agr activity in CC398 strains. In addition, the secretome profiles revealed also an abundant core secretome of *S. aureus* virulence factors that is conserved across lineages.

Out of the 869 identified proteins, 50 secreted virulence factors are the ones most abundantly found in all secretomes and contribute with 86.5% to the core secretome (Fig. 4*C*). These 50 most abundantly secreted proteins include 24 extracellular, 12 cell surface-associated, 2 membrane and 10 cytoplasmic proteins. Moreover, among the top 10 secreted extracellular proteins are the main cytolytic toxins (Hla, Hlb, PSMβ1), the major autolysins (Atl, IsaA, Sle1), lipases and lipoteichoic acid hydrolases (Lip, Geh, LtaS) and cysteine proteases (SspB) that contribute to 59.2% of the total secretome. The normalized protein abundances of all secreted proteins are displayed in Voronoi secretome treemaps which visualize the contribution of these top ten extracellular proteins and surface factors to the main part of the secretome in all isolates (Fig. 5). In the treemaps, the cell size denotes the average 99 percentile normalized protein abundance of the proteins in all strains. The color code indicates the 99-percentile normalized protein abundance in each single *S. aureus* isolate. Thus, the treemaps visualize the normalized amounts of the secreted proteins in each strain (by color-code) and as average in all *S. aureus* isolates (by cell size) (Fig. 5). The secretome treemaps indicate that most extracellular and cell-surface proteins are most abundant and conserved in the core secretome while smaller differences are displayed especially in the category of accessory proteins and cellular metabolism between the three lineages. The secretome profiles were phylogenetically clustered revealing lineage-specific groups. The differences in the secretomes between the three genetic lineages are described in the next sections.

##### Comparison of the CC8, CC22 and CC398-specific Protein secretion Patterns

Next, we analyzed the differences in the secretion of virulence factors between human and livestock lineages. The Venn diagram in Fig. 3*A* shows that out of 869 secreted proteins, the majority of 538 proteins are common in all isolates and are part of the core secretome (supplemental Tables S6, S7). The CC-specific differences in the secretomes represent 5–10% of all secreted proteins. In total, 37 proteins were quantified only in CC22-isolates, 96 proteins are CC398-specific and 54 proteins appeared to be CC8-specific. The isogenic MRSA/MSSA pair SK41/SK42 share 57 proteins in their secretomes. In addition, 19 and 6 proteins were exclusively found in SK41 and SK42, respectively. Together these data show that the majority of secreted proteins are common in CC398, CC8, and CC22. A subset of exoproteins appears to be exclusively secreted in one clonal complex or in specific strains (supplemental Tables S6, S8), which are described in the following section.

##### The Spl Proteases, Superantigens and The Epidermin Protease Are CC8 or CC22-specific Secreted Proteins and Not Encoded in the CC398 Genomes

In total, 109 secreted proteins can be allocated to the accessory genome (supplemental Tables S6 and S8). Many of those are phage-related proteins, enterotoxins, surface factors, immune evasion cluster proteins, proteases and hypothetical proteins that are encoded and secreted only in few strains. Among the virulence factors exclusively secreted in CC8 isolates are the serine protease-like proteins SplA, SplB, SplC, SplD, and SplE, which are encoded by the *spl* operon present on the νSaβ pathogenicity island (Table II; supplemental Tables S6, S8). The *spl* operon is only encoded in the CC8 genomes. In addition, secretion of EpiP was quantified in the two CC8 isolates because the epidermin biosynthesis operon is absent in the genomes of CC22 and CC398 isolates (Table II; supplemental Tables S6, S8). The epidermin leader peptide processing serine protease (EpiP) is a lantibiotic processing protease recently characterized in *S. aureus* (81).

Moreover, the pyrogenic superantigens, such as staphylococcal enterotoxins (SE A,B,C,D,E,I,P) and SE-like proteins (Ssl) are known to be almost absent in CC398 isolates (12). The high variation among the genes encoding superantigens in CC8 and CC22 isolates and their partial absence in CC398 could be confirmed in both proteogenome and secretome comparison (Table II; supplemental Tables S3, S5, S8). In total, 10 Ssl proteins and 19 enterotoxins are annotated in the genomes of the 18 isolates (supplemental Table S5). Six Ssl proteins were detected in all secretomes of the 8 isolates at low levels. Ssl12 and Ssl13 were only secreted in CC8 and CC398 because the genes are absent in CC22 genomes. Similarly, eight enterotoxins are strain-specific and could be quantified only in few secretomes of CC8 or CC22 isolates. The enterotoxins Sei, Sem, Sen, and Yent2 were quantified exclusively in CC22 isolates whereas Sep and homologs of EntA, EntD and EntG (RKI316, RKI85, RKI350) were secreted at high levels only in specific CC8 strains (Table II; supplemental Tables S5, S8). CC8 strain 38879 seems to be a hyperproducer of enterotoxins, which secretes high levels of the EntA, EntD, EntG homologs and SelX. Staphylococcal enterotoxins are in part encoded by the enterotoxin gene cluster (*egc*) that is located on the pathogenicity island SaPI3 (82, 83). Overall, these 8 enterotoxins detected in the secretomes of specific CC8 and CC22 strains are not encoded in the CC398 genomes. In addition, the cell wall protein SasG is not secreted in CC398 strains whereas FnbB2 is only found in the CC398 secretomes and genomes.

##### Agr-controlled cell-surface proteins and autolysins are secreted at significantly lower levels in CC398 isolates

**Fig. 6.**
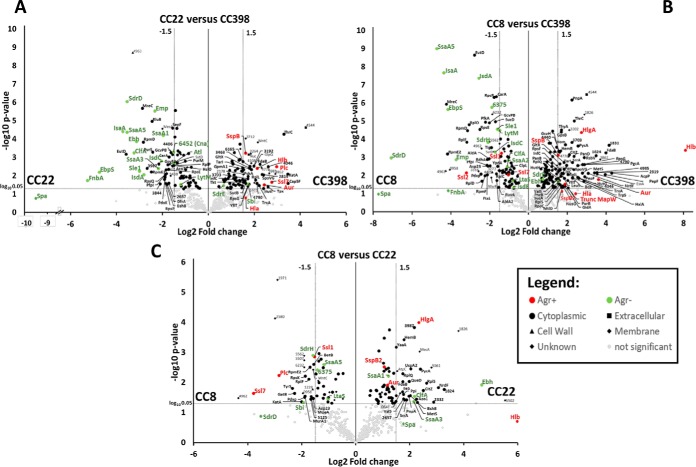
**Volcano Plots of differentially secreted proteins in the secretomes between CC8, CC22, CC398.** The log2 fold changes of >+1.5 and <−1.5 (*p* value <0.05) are shown as significant differences in the average abundance of secreted proteins in the genetic lineages CC22 *versus* CC398 (*A*), CC8 *versus* CC398 (*B*) and CC8 *versus* CC22 (*C*) as calculated in supplemental Tables S7*A*–S7*E*. Only secreted proteins were considered that were identified in all 3 clonal complexes to reveal regulatory secretome differences. Although Agr-controlled surface-associated proteins were significantly down-regulated in CC398 isolates (green spots), exotoxins (Hla, Hlb) and extracellular enzymes (Aur, SspB, SspB2) were up-regulated in CC398 *versus* CC22 and CC8 (red spots). More cytoplasmic proteins are secreted in CC398 *versus* CC8 (black spots). Other significantly differentially secreted proteins were labeled with black symbols according to their sub-cellular localization as given in the legend. Non-significantly differentially secreted proteins between the CCs are gray spots.

Next, we aimed to identify CC-specific secreted virulence factors and cell surface proteins that are common in the core proteogenome, but differentially secreted because of regulatory differences across the CCs. Volcano plots were used to reveal statistically significant differences with a log2 fold-change cut-off of ± 1.5 and *p* values of <0.05 in the levels of commonly secreted proteins between CC8, CC22, and CC398 isolates. Overall, the levels of 117, 87, and 59 proteins were significantly different between the secretomes of CC398/CC8, CC398/CC22 and CC22/CC8, respectively (Fig. 6*A*–6*C*, supplemental Tables S7*A*–S7*E*). Compared with LA-MRSA CC398, the secretomes of CC22 and CC8 isolates are more closely related which correlates with the phylogeny of our proteogenome treemap (Fig. 2).

The Volcano plots visualize the regulatory differences in the secreted virulence factors between livestock-associated CC398 and the human specific lineages CC8 and CC22 that can be linked to different Agr activities (Fig. 6*A*–6*C*). The AgrAC quorum-sensing system up-regulates genes for toxins (*e.g. hla, hlb, psms*) and down-regulates genes encoding surface factors (*e.g. spa, fnbA*) during the stationary phase (45, 46, 84–87). Thus, it was intriguing that 12, 15, and 8 surface factors and autolysins were secreted at 1.5–8 log2-fold lower amounts in CC398 isolates compared with CC8, CC22 and both, respectively (Fig. 6*A*–6*C*, supplemental Tables S7*B*–S7*E*). These significantly differentially secreted surface proteins in CC398 *versus* CC8 or CC22 include many MSCRAMMs and SERAMs, such as secretory extracellular matrix and plasma binding protein (Emp), elastin-binding protein (EbpS), fibrinogen-binding proteins (Ebh, ClfA, and FnbA), collagen adhesion (Cna), the surface protein (SasG), Ser-Asp rich fibrinogen/bone sialoprotein-binding proteins (SdrD, SdrH), iron-regulated surface determinants (IsdA, IsdC), lytic cell wall hydrolases and autolysins (SceD, IsaA, Sle1, Atl), secretory antigens (Ssa1, Ssa5) and the IgG-binding protein A (Spa) (Fig. 6*A*–6*C*, supplemental Tables S7*A*–S7*E*). Of note, the *p* values of Spa and FnbA are lower because of strain-specific expression differences across the CCs. The differences in the secretion of surface-associated proteins between human and zoonotic isolates are not linked to genomic differences. The lower secretion of surface-associated proteins in CC398 agrees with previous studies (29). Our results suggest a higher Agr activity in all CC398 isolates resulting in stronger repression of cell surface factors compared with CC8 and CC22.

##### Positively Agr-controlled α- and β-hemolysins (Hla, Hlb) and Extracellular Enzymes Are Secreted At Higher Levels in CC398 Isolates

The secreted proteins of significantly higher abundance in CC398 isolates (log2 fold-change >1.5) compared with CC22 or CC8 include the α-, β- and γ-hemolysins Hla, Hlb and HlgA, truncated MapW, glycerophosphoryl diester phosphodiesterase GlpQ, the aureolysin Aur, the cysteine protease SspB and the hyaluronate lyase (SAUPAN004544000) (Fig. 6*A*–6*C*, supplemental Tables S7*A*–S7*E*). Most of these toxins and extracellular enzymes are positively regulated by the Agr system which is important for virulence (45, 84, 87, 88).

Secretion of Hla was 1.6–2.4 log2-fold increased in CC398 strains, but strain-specific variations were observed across the CCs which explains the lower *p* value. Moreover, Hla and Hlb were secreted in high amounts and are among the top ten secreted proteins in the selected four CC398 isolates (Fig. 5–6; Table II; supplemental Tables S6–S8). The toxin β-hemolysin (Hlb), also known as sphingomyelinase was secreted as full-length intact protein at 8 log2-fold higher abundance in CC398 compared with CC8 isolates. The majority of human CC8 and CC22 isolates encode non-functional truncated Hlb variants because of the prophage ΦSa3 disrupting the *hlb* locus (30–32). Thus, we neither detected Hlb secretion in the CC8 strains 38879 and 38880, nor in the CC22 strain 18787. Only low level of truncated Hlb was found in the secretome of the CC22 isolate 24749 (Fig. 5; Table II; supplemental Tables S6–S8). The truncated Hlb variants in all CC8 and CC22 isolates (except for 38881) are shown in the alignment of Hlb protein sequences (supplemental Table S4, supplemental Fig. S3). Because of the lack of ΦSa3 integration into the *hlb* locus, the full-length Hlb protein was secreted at high levels only in the four selected CC398-isolates (Fig. 5; Tables S6-S8). Overall, the elevated secretion of toxins (Hla, Hlb) and many extracellular enzymes (Aur, SspB, GlpQ, SAUPAN004544000) further points to an enhanced Agr activity in CC398 isolates.

##### Secretion of subsets of cytoplasmic proteins is increased in CC398 isolates versus CC8

Among the differentially secreted proteins are also many cytoplasmic proteins (CPs) which are labeled as black spots in the Volcano plots (Fig. 6; supplemental Tables S7*A*–S7*E*). Secretion of CPs is observed in all previous secretome studies in different bacteria, but the mechanisms are unknown (89–92). The majority of secreted CPs are involved in energy metabolism, biosynthetic pathways, antioxidant functions and protein translation (supplemental Tables S6–S8). Recent studies have revealed a role of the phenol-soluble modulins (PSMα) in excretion of CPs through membrane damage resulting in the release of CPs, lipids, nucleic acids and ATP (93). Because of their small size, we likely missed PSMα toxins in the secretomes, whereas strong PSMβ1 secretion was observed in all isolates at varying levels. Because PSMs are highly cytolytic and their expression is under strict Agr-control (46, 94), we analyzed the secretion differences of CPs between CC398 *versus* CC22 and CC8. The Volcano plots showed that a subset of 56 CPs are secreted at higher amounts in the secretomes of CC398 isolates *versus* CC8 strains, whereas 70 CPs are secreted below the log2 fold-change cut-off of 1.5 and 26 CPs are secreted at significantly decreased levels in CC398 (Fig. 6*A*, supplemental Table S7B). However, no significant differences in the levels of CPs were observed between CC3398 and CC22 isolates. Specifically, 25 CPs are present at increased levels in the secretomes of CC398 whereas other 25 CPs are secreted at lower amounts and 91 are similar abundant in both CC398 and CC22 exoproteomes. This can be explained by the CC22/CC8 secretome differences, because 22 CPs were more secreted in CC22 isolates compared with only 12 CPs secreted at lower levels in CC22 *versus* CC8 (Fig. 6*C*, supplemental Table S7*C*). Altogether, our data revealed a ∼2-fold enhanced secretion of a subset of CPs in CC398 isolates *versus* CC8 which agrees with the predicted higher Agr activity. However, quantification of cell lysis during the stationary after 9 h of growth using survival assays (CFUs) and live-dead staining of cells did not reveal a difference in cell viability and no increased lysis during the stationary phase across all strains (supplemental Fig. S2).

##### Hemolytic Activity Is Strongly Increased in CC398 Compared With CC22 and CC8 Isolates

To further investigate the phenotypes of CC398, CC22, and CC8 isolates, we assessed their hemolytic activities and biofilm formation which are important for pathogenesis and controlled by the Agr system and SigB (43, 95).

**Fig. 7.**
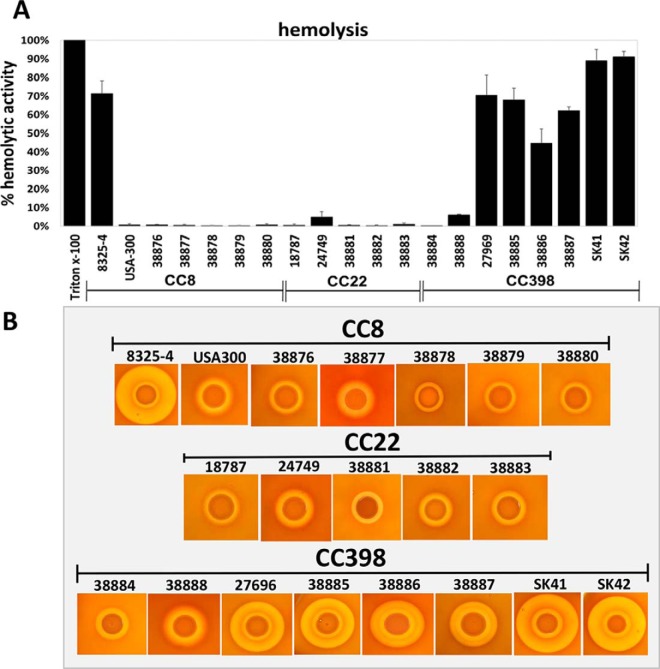
**Hemolytic activities of *S. aureus* isolates of CC8, CC22 and CC398 indicate increased α- and β-hemolysis in CC398.** Extracellular fractions were harvested from 18 *S. aureus* during the stationary phase after 16 h of growth. Total hemolytic activities of filtered culture supernatants were analyzed with 2% sheep blood erythrocyte solution. *A*, Lysis of erythrocytes was measured as absorbance at 405 nm. Hemolysis was normalized to the Triton-X100 positive control, which was considered as 100% hemolysis. Secreted proteins of the CC398 isolates displayed much higher hemolytic activities than CC8 and CC22 isolates. The increased hemolysis in most CC398 strains (except for 38884 and 38888) correlates with intact β-hemolysin expression due the absence of the β-hemolysin converting prophage ΦSa3. The phage ΦSa3 disrupts the *hlb* locus in the majority of CC8 and CC22 isolates (except for 38881). Strains 38881 and 38884 have intact *hlb*, but are *agrC*-defective. In addition, elevated α-hemolysin expression in CC398 contributes to strong hemolysis. The results are presented as average values of 5–10 independent biological experiments with measurements made in duplicates. Error bars represents S.E. *B*, The β-hemolysis can be visualized after spotting of 5 μl bacterial suspension on sheep blood agar plates as shown previously (77). Strong β-hemolytic rings can be visualized in most CC398 isolates except for strain 38888. The *agr*-defective strains 38881 and 38884 show only small β-hemolysis.

The hemolytic activities of the supernatants from all 18 *S. aureus* isolates were analyzed in quantitative sheep blood hemolysis assays (Fig. 7*A*). Much higher hemolytic activities could be observed for six CC398 isolates compared with CC8 and CC22 isolates. The hemolytic activities of CC8 and CC22 were very low. The highest hemolytic activities were measured for the CC398 MRSA/MSSA pair SK41 and SK42 with values of 89–91.1%. Four human CC398 isolates also showed high hemolytic activities of 40.9–70.4%. These results confirmed the enhanced Hla and Hlb secretion in the CC398 secretomes compared with CC8 and CC22 (Fig. 5 and 6; Table II, supplemental Tables S6–S8). The δ-hemolysin (Hld) was secreted at low levels in the secretomes and not significantly different between the CCs (supplemental Tables S7–S8) suggesting that the strong hemolysis in CC398 is mainly caused by Hla and Hlb activities. β-hemolysin activity of the extracellular fractions was further confirmed on sheep blood agar plates for six CC398 isolates (Fig. 7*B*) (76, 77). The remaining CC398 strains 38884 and 38888 showed only a low hemolysis in the quantitative assays (Fig. 7*A*). Strain 38884 contains an IS256 transposase gene which disrupts the *agrC* gene resulting in truncated nonfunctional AgrC as previously reported in *S. aureus* T031 and HC1335 (96, 97). CC398 strain 38888 is IEC positive carrying the prophage ΦSa3 which disrupts the *hlb* gene. Overall, the hemolysis results support that the high hemolytic activities of most CC398 isolates are caused by increased Hla and Hlb secretion as quantified in the secretome.

##### CC398 Isolates Show Reduced Biofilm Formation, Less Pigmentated Macrocolonies and Lower Staphyloxanthin Levels

**Fig. 8.**
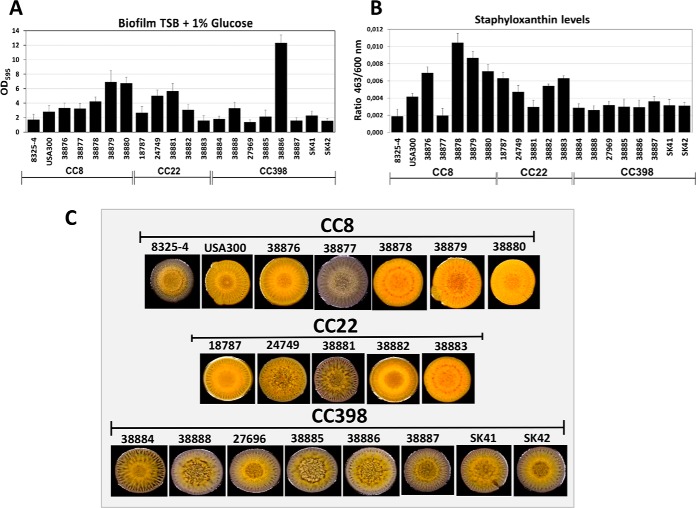
**Biofilm formation, staphyloxanthin levels and structured macrocolony phenotypes of 18 *S. aureus* isolates of CC8, CC22 and CC398.** *A*, Biofilm formation was analyzed from overnight cultures that were diluted to an OD_580_ of 0.5 in TSB with 1% glucose. 200 μl cells were transferred in triplicate to microtiter plate wells for attachment. The attached biofilm cells were stained using 0.1% crystal violet solution in the microtiter plate wells and resuspended with 0.1% SDS for quantitative measurements of the biofilm crystal violet solution at an OD_595_. For comparison, the laboratory CC8 strains *S. aureus* 8325–4 and USA300 were included in the biofilm assays. *B*, Staphyloxanthin levels were measured from cell pellets of 1 ml overnight cultures after methanol extraction as absorbance at 463 nm and normalized to the OD_600_ of the cell culture (463/600 nm ratio). *C*, Structured macrocolony formation and staphyloxanthin pigmentations of the strains was analyzed by spotting 2 μl cell suspension on TSB-agar with 100 mm MgCl_2_ for 5 days. *S. aureus* CC398 isolates with increased α- and β-hemolysin secretion showed lower ability for biofilm formation (*A*), lower staphyloxanthin levels (*B*) and white-yellow pigmented macrocolonies (*C*) because of reduced SigB activity. The results of the biofilm and staphyloxanthin measurements in (*A, B*) are shown as average values of 5 independent biological experiments. Error bars are S.E.

Hemolysis and biofilm formation are oppositely controlled by the Agr system because cytolytic toxins are induced and cell wall proteins are repressed at the same time during the stationary phase (45, 84, 86, 87, 98). Thus, Agr down-regulates biofilm formation through repression of surface proteins, such as Spa and other MSCRAMMs that are responsible for attachment. The reduced secretion of many cell-surface proteins in the secretomes of CC398 isolates might suggest a reduced ability for biofilm formation. Thus, we investigated biofilm formation of the 18 isolates using microtiter plate crystal violet assays and macrocolony phenotypes (Fig. 8*A*, 8*C*). The results of the crystal violet assays are quantified as OD_595_ values. The strongest biofilm formations in the crystal violet assays were observed for the CC398 isolate 38886 (OD_595_ of 12.3), for the CC8 isolates 38879 and 38880 (OD_595_ of 6.9 and 6.7), and for CC22 isolates 24749 and 38881 (OD_595_ of 5 and 5.7) (Fig. 8*A*). All remaining isolates showed lower biofilm formations including the laboratory strains *S. aureus* 8325–4 and USA300 (OD_595_ of 1.7–2.5). Interestingly, six CC398 strains (except for 38886 and 38888) showed much lower levels of biofilm formation compared with CC8 and CC22 strains. These data correlate with the decreased levels of cell surface proteins in the secretome of the CC398 isolates that might contribute to decreased surface attachment. Thus, biofilm formation appears to be decreased in most CC398 isolates. The genes associated with the high biofilm formation in 38886 remain to be elucidated.

Next, we analyzed the differences in macrocolony biofilm phenotypes on MgCl_2_-TSB agar plates for all 18 isolates of CC8, CC22 and CC398 (Fig. 8*C*). Macrocolony formation was previously shown to require the alternative sigma factor SigB which inhibits the Agr system. SigB also controls the *crt* operon for staphyloxanthin biosynthesis as yellow antioxidative pigment (40–44, 98). Interestingly, most CC8 and CC22 isolates showed stronger pigmentation compared with CC398 isolates. Exceptions with lower pigmentation were the *agrC*-defective strain 38881 and strain 38877 with increased α-hemolysis (Fig. 8*C*). The lower staphyloxanthin levels in most CC398 isolates were also quantified after methanol extraction from cell pellets (Fig. 8*B*). In conclusion, the higher hemolytic activity, lower biofilm formation and reduced staphyloxanthin production in CC398 isolates might be related to decreased SigB activity in CC398 strains.

##### Northern Blot Analysis Confirm Higher Agr Activity and Lower SigB Activity In CC398 Isolates

**Fig. 9.**
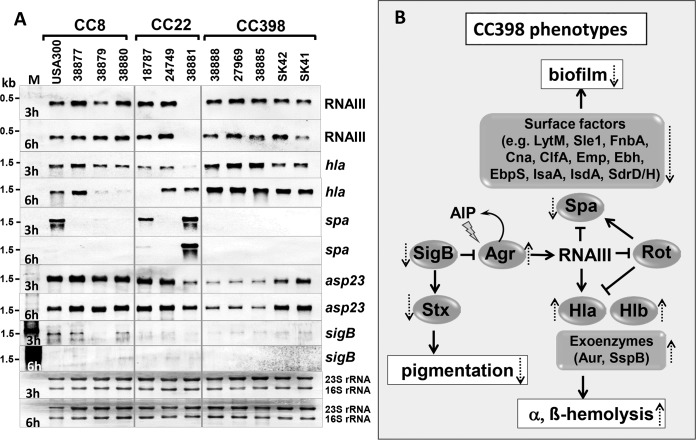
**Transcriptional analyses indicate differential Agr and SigB activities in human and zoonotic *S. aureus* isolates.** *A*, For Northern blotting analyses RNA was isolated of selected *S. aureus* CC8, CC22 and CC398 isolates grown in TSB medium and harvested during the exponential growth and stationary phase after 3 and 6 h of growth. Agr and SigB activities were analyzed using digoxygenin-labeled RNA probes specific for RNAIII, *hla* and *spa* (Agr), *asp23* and *sigB* (SigB). The methylene-blue stained bands of the 16S and 23S rRNAs in the Northern blots are shown as loading controls at the bottom. The Northern blotting experiments were performed in 3 biological replicate experiments. *B*, Schematics of the Agr and SigB regulatory network for expression of secreted toxins, exoenzymes, surface factors that are involved in hemolysis, biofilm formation and staphyloxanthin production in *S. aureus*. CC398 isolates showed increased *hla* transcription and decreased *asp23* and *sigB* operon transcription compared with CC8 or CC22 strains. This indicates lower SigB activity and increased Agr activity in CC398 strains resulting in higher α- and β-hemolysis as well as lower surface factors, biofilm production and staphyloxanthin amounts. Positive and negative regulations are indicated. Broken arrows show induction or repression of virulence factors and phenotypes in CC398 strains as revealed in this study.

To further investigate the differences in Agr and SigB regulation, Northern blotting transcriptional analyses were performed using RNA isolated during the exponential growth and stationary phase after 3 and 6 h of growth (Fig. 9*A*). Hybridizations were performed with RNA probes for hallmark genes of Agr (RNAIII, *hla, spa*) and SigB (*asp23*, *sigB*) regulation. Surprisingly no significant differences in the RNAIII levels were observed between most isolates. Strain 38881 was confirmed as *agrC*-defective because RNAIII is absent. However, *hla* transcription was significantly increased in most CC398 isolates especially during the stationary phase after 6 h of growth. As expected, *spa* transcription was strongly repressed by RNAIII in most isolates, but not in the *agrC*-defective 38881 because of missing RNAIII. Interestingly, we could confirm a decreased transcription of the SigB-dependent *asp23* and *sigB* genes and operons in most CC398 isolates, only SK41/SK42 showed higher *asp23* transcript levels. Asp23 is present also at lower levels in the CC398 secretomes (supplemental Tables S7–S8). The Northern blotting results indicate that SigB activity is reduced in CC398 isolates which is agreement with their decreased staphyloxanthin levels (Fig. 8*B*).

## DISCUSSION

Previous secretome analysis of *S. aureus* patient isolates revealed 63 secreted proteins, including only 7 common, such as autolysins (Atl, IsaA, LytM) and lipase (Lip). Other secreted proteins were found at least in 80% of these isolates, including Aur, Geh, GlpQ, LtaS, Hla, HlgB, SA0570, SA1812, SspA and SspB and can be defined as core secretome (39). A similar core set of shared secreted virulence factors was found in 17 bovine mastitis isolates confirming the most abundant virulence factors among human and animal *S. aureus* isolates (99). In this study, the extracellular enzymes (Lip, Geh, SspB, Nuc, Aur, LtaS), autolysins (Atl, IsaA, Sle1) and toxins (Hla, Hlb) also account for 59.2% of the pan-secretome of eight isolates from human and pigs of CC8, CC22 and CC398 (Figs. 4–5, Table II, supplemental Table S8).

In total, we identified 869 proteins in the secretomes, including 64 secreted virulence factors and 37 surface-associated proteins that are autolysins or adhesins binding to host matrix components (supplemental Table S6). Moreover, the calculation of protein abundances together with PSORTb prediction revealed that the majority of the 50 most abundantly secreted and surface proteins are predicted to be in the extracellular or cell wall compartments and released into the supernatant. These predicted extracellular proteins account for 86.5% of the secretome and are largely conserved in most of the isolates. Thus, this secretome study expands previous exoproteome comparison to elucidate the quantitive contribution of secreted virulence factors and surface-associated proteins to the core secretome of *S. aureus*.

Previous secretome studies also revealed a remarkable high exoproteome heterogeneity and plasticity across *S. aureus* patient and bovine isolates that are only partly related to genomic variations, but also to regulatory differences (39). In this work, we quantitatively explored the heterogeneity of secreted virulence factors resulting from genomic and regulatory differences across the dominant human and zoonotic lineages CC8, CC22 and CC398. CC398 is of interest because of a debate about its pathogenicity because many important toxins are missing in the majority of CC398 isolates, such as PVL and superantigens. However, CC398 isolates have been responsible for severe systemic human infections and have a strong cytolytic effect on human neutrophils and epithelial cells because of their high level secretion of α- and β-hemolysins (12, 29, 100). We could confirm here that four CC398 strains secrete higher levels of intact α- and β-hemolysins. In contrast, the majority of CC8 and CC22 isolates encode truncated Hlb variants because of the integration of prophage ΦSa3 into the *hlb* locus (29, 101) (supplemental Fig. S3). The enhanced β-hemolysin secretion in most CC398 isolates contributed to the strong β-hemolysis in sheep blood assays that was not observed in the human-specific CC8 or CC22 isolates.

Using Volcano plots and secretome treemaps, we demonstrated quantitatively different expression levels of secreted proteins and surface factors between human lineages (CC8, CC22) and livestock strains (CC398) that are mediated by both, genome differences and regulatory differences because of Agr and SigB activities. The genomic differences could be related to the Spl-proteases, the epidermin processing protease EpiP and the superantigen-diversity which are human CC8 or CC22-specific factors, absent in the CC398 genomes.

The serine proteases SplA, SplB, SplC, SplD, and SplE are encoded on the νSaβ pathogenicity island that is present in CC8 clones only. SplA can cleave mucin produced by human lung cells and Spl proteins have been shown to cause disseminated lung damage in a rabbit model of pneumonia (102). This suggests the importance of these proteins in *S. aureus* host tissue invasion and spreading. Spls are immunogenic and elicit IgE antibody responses in asthmatic patients (103). However, the functions of the Spl proteases in *S. aureus* are unknown and it might be possible that they have human-specific substrates (102).

The epidermin lantibiotic biosynthesis is specifically encoded in the genomes of CC8 isolates. EpiP is involved in the maturation of the antibiotic epidermin after its release in the extracellular milieu (104). In addition, EpiP can cleave collagen and casein suggesting that it could play a role in *S. aureus* colonization (104).

The absence of the majority of superantigens and enterotoxins in CC398 isolates was further confirmed in our secretome study (12, 29). The lack of secretion of the superantigens SEC-bov, SEL and TSST in bovine mastitis isolates has been previously observed (99). In this work, the diversity and expression of enterotoxins in CC8 and CC22 isolates was highly strain-specific. Sei, Sem, Sen and Yent2 were specific for CC22 isolates. Instead, SelX and homologs of EntA, EntD and EntG (RKI316, RKI85, RKI350) were secreted at high levels in the CC8 strain 38879 (Table II; supplemental Tables S6–S8). In our study, superantigens seem to be human-specific CC8 and CC22 virulence traits, although other studies have reported their presence in bovine mastitis isolates (105). Superantigens stimulate T-cell proliferation to induce massive cytokine induction, which can lead to toxic shock syndrome, as shown for TSST and SEC (106). In addition, the low expression of six superantigen-like proteins (Ssls) confirms previous results (107). Ssl proteins do not possess superantigenic activity but target several key elements of the host innate immune system (107).

Additionally, we found regulatory differences between CC8, CC22, and CC398 in the secretion of surface proteins, α- and β-hemolysins as well as extracellular enzymes. The Volcano plots showed significantly decreased secreted surface factors including MSCRAMMs (FnbA, SasG, EbpS, SdrD) and SERAMs (Emp, Efb, Spa, SceD, and IsaA). The surface factors LytM, EbpS and Spa were also secreted at lower levels in bovine mastitis isolates (99). In contrast, the secretion of α- and β-hemolysins and other extracellular enzymes was significantly enhanced in the CC398 isolates. Virulence factors are controlled by the Agr system via the RNAIII which positively regulates toxins and extracellular proteases and negatively regulates surface proteins during the stationary phase (45, 46, 84–87). The alternative sigma factor SigB inhibits Agr activity and promotes biofilm and macrocolony formation (Fig. 9*B*) (40, 42, 43). Quantification of the transcript levels supported the higher *hla* expression in CC398 strains whereas SigB-dependent *asp23* transcription was decreased. Thus, the lower secretion of surface proteins and higher Hla and Hlb secretions in the CC398 clones are probably caused by higher Agr activity because of reduced SigB activity in CC398 compared with CC8 and CC22 strains. A decreased adherence of CC398 isolates to plasma fibrinogen and human cells was previously reported which might be related to lower expression of MSCRAMMs (29, 108). The decreased SigB activity was also evident by the reduced pigmentation of macrocolony phenotypes and lower staphyloxanthin levels in CC398 isolates. Collectively, our secretome and transcriptional data indicate that a decreased SigB activity in CC398 causes higher Agr activity resulting in increased α- and β-hemolysis as well as decreased biofilm formation and staphyloxanthin production. Thus, our secretome data and virulence phenotypes identify Agr and SigB as key players for regulatory differences in virulence factor secretion between epidemic human and zoonotic CCs.

Another aim of this study was to identify new virulence determinants in the secretomes of CC398 that mediate enhanced animal colonization (28). Our results suggest that CC398 isolates might compensate for the lack of superantigens and surface factors by the strong cytotoxic effect of α- and β-hemolysins and overexpression of extracellular enzymes. Because of the higher Agr activity in CC398 strains, we identified significantly higher levels of several extracellular enzymes in the secretomes of CC398 strains (supplemental Fig. S6, supplemental Table S7E), including aureolysin (Aur), hyaluronate lyase (SAUPAN004544000), glycerophosphoryl diester phosphodiesterase (GlpQ) and two cysteine proteases (SspB1, SspB2). Aureolysin plays an important role for staphylococcal immune evasion by cleavage and inhibition of the complement factor C3 (109). Hyaluronate lyase also contributes to virulence of *S. aureus* (110). GlpQ was shown to degrade glycerophosphodiester head groups of human phospholipids, such as glycerophosphocholine to generate glycerol-3-phosphate as carbon and phosphate source (111). GlpQ was required for growth of *S. aureus* in the presence of glycerophosphocholine (111). The staphylococcal cysteine proteinase staphopain B (SspB) cleaves CD11b and CD31 on the surface of macrophages and neutrophils to induce phago-cytosis resulting in depletion of phagocytes (112–114). In future studies the roles of these virulence factors should be analyzed regarding host specificity of CC398 isolates.

We further identified about 70% of secreted CPs in the pan-secretome that contribute, however, with only 12.9% to total secretome abundance (Fig. 4*B*). Thus, the secreted levels of CPs were much lower compared with predicted secreted or cell-wall associated proteins in the pan-secretome. This non-classical protein secretion of the CPs is most likely not because of cell lysis and was shown to require the Agr-controlled PSMα toxins which damage cytoplasmic membranes resulting the cell leakage (90, 93). We could show that secretion of 56 CPs is ∼2-fold enhanced in CC398 isolates *versus* CC8 which is in accordance to the higher Agr activity of CC398. However, cell lysis was not increased in CC398 strains compared with CC8 or CC22. This may indicate a fine-tuned export of subsets of cytoplasmic proteins because of differential Agr activities that does not lead to an increased cell lysis. Moreover, the levels of secreted CPs with antioxidant functions were increased in CC398 isolates (*e.g.* TrxA, SAUPAN002819000, KatA). This could indicate a higher resistance to oxidative stress under infection conditions or a compensatory mechanism to the lower staphyloxanthin levels in CC398 isolates which remains to be further investigated.

In conclusion, our combined secretome and phenotype results have identified lower SigB activity and higher Agr activity in CC398 isolates as main regulatory difference between human and zoonotic *S. aureus* isolates (Fig. 9*B*). Reduced SigB activity in CC398 results in higher α- and β-hemolytic activities, reduced biofilm formation and lower staphyloxanthin levels. However, the SigB-dependent factor required for repression of Agr activity is not known and requires further work. In addition, we could not show a higher RNAIII transcription in CC398 compared with CC8 and CC22 isolates although the secretome differences clearly support higher Agr activites in CC398 strains. Thus, additional regulatory factors or promoter mutations could be involved in post-transcriptional regulation of genes encoding toxins, extracellular enzymes and surface factors in CC398 strain that remain to be elucidated.

## DATA AVAILABILITY

The mass spectrometry data of the secretomes of the eight *S. aureus* isolates of CC8, CC22 and CC398 have been deposited to the ProteomeXchange Consortium via the PRIDE (74) partner repository (https://www.ebi.ac.uk/pride/archive/) with the dataset identifier PXD008797. The MS/MS spectra of all identified peptides have been deposited to the MS Viewer database (http://msviewer.ucsf.edu/prospector) under the key r1mmcmhlpi. The Whole Genome Shotgun project of the genomes sequences of the eight CC398 isolates 27969, 38884, 38885, 38886, 38887, 38888, 38951-SK41, 40439-SK42 has been deposited at DDBJ/ENA/GenBank (https://www.ebi.ac.uk/ena) under the accession numbers QBFK00000000, QBFL00000000, QBFM00000000, QBFN00000000, QBFKO0000000, QBFP00000000, QBFQ00000000, QBFR00000000.

## Supplementary Material

supplemental Table S6

## Footnotes

^1^ The abbreviations used are:

MRSA: methicillin-resistant *Staphylococcus aureus*
CA-MRSA: community-acquired MRSA
CHIPS: chemotaxis inhibitory protein
CC: clonal complex
CPs: cytoplasmic proteins
ENA: European Nucleotide Archive
HA-MRSA: hospital-acquired MRSA
HGT: horizontal gene transfer
Hla: α-toxin or α-hemolysin
Hlb: β-hemolysin or sphingomyelinase
HPLC: high-pressure liquid chromatography
IMT: Institute of Microbiology and Epizootics
IEC: immune evasion cluster
LA-MRSA: livestock-associated MRSA
LB: Luria broth
LC-MS/MS: liquid chromatography tandem mass spectrometry
LFQ: Label-free quantification
LTQ: linear trap quadrupole
MGE: mobile genetic elements
MLST: multi-locus sequence typing
MSCRAMM: microbial surface components recognizing adhesive matrix material
MSSA: methicillin-sensitive *Staphylococcus aureus*
MS: mass spectrometry
MS/MS: tandem mass spectrometry
NaOCl: sodium hypochlorite
NEM: N-ethylmaleimide
OD_580_: optical density at 580 nm
PAN-ID: SAUPAN-number according to Aureowiki database
PSM: Phenol-soluble modulin
PVL: Panton-Valentine leukocidin
RKI: Robert Koch Institut
SAK: staphylokinase
SCC*mec*: *Staphylococcus* Cassette Chromosome *mec*
SCIN: staphylococcal complement inhibitor
SE: food-poisoning staphylococcal enterotoxins
SERAM: secretable expanded repertoire adhesive molecules
ST: sequence type
TCA: trichloroacetic acid
TSB: Tryptic soy broth
TSST: toxic shock syndrome toxin.
