# Supplementary material for: Comparative Secretome Analyses of Human and Zoonotic Staphylococcus aureus Isolates CC8, CC22, and CC398
Source: Mol Cell Proteomics. 2018 Sep 10;17(12):2412–33. doi: 10.1074/mcp.RA118.001036 (PMC6283302; doi:10.1074/mcp.RA118.001036)
Supplement: supplemental Table S6 [file RA118.001036_index.html]

Supplement to Comparative secretome analyses of human and zoonotic Staphylococcus aureus isolates of CC8, CC22 and CC398 | Molecular & Cellular Proteomics

## Supplemental Data

- Figure S1 - MS/MS Spectra of proteins with only 1 identified unique peptide in all strains and 2/3 of replicates.
- Figure S2 - Growth curves (A), survival counts (B) and live-dead assay (C) of S. aureus strains of CC8, CC22 and CC398 during the stationary phase
- Figure S3 - Alignment of &#x00DF;-hemolysin protein variants as revealed by the FASTA protein sequences that are translated from the genome sequences of the selected S. aureus isolates from CC8, CC22 and CC398
- Tables S1 and S2 - Table S1: List of all peptide identifications in the 8 S. aureus isolates of CC8, CC22 and CC398. Table S2A: List of all unsorted original protein identifications in the 8 S. aureus isolates. Table S2B: List of all sorted protein identifications in the 8 S. aureus isolates including false identification and contaminants. Table S2B includes the information on unique peptides, % sequence coverage, peptide identification type and LFQ intensity values. Table S2C: Final list of 869 protein identifications in the 8 S. aureus isolates. Table S2C includes the information on unique peptides, % sequence coverage, peptide identification type and LFQ intensity values of the 869 protein IDs listed in Table S6.
- Table S3 - Pan-proteogenome of 3487 unique protein IDs present in 18 S. aureus isolates of CC8, CC22 and CC398 without allele variants.
- Table S4 - Pan-proteogenome of 3820 unique protein allele variants annotated in 18 S. aureus isolates of CC8, CC22 and CC398
- Table S5 - Virulence factors present in 18 S. aureus isolates of CC8, CC22 and CC398
- Table S6 - Identification of 869 secreted proteins in the secretome of the eight S. aureus isolates of CC8, CC22 and CC398
- Table S7 - Table S7A: Quantification of the log2-fold changes in the secreted amounts of 869 proteins between CC398/CC8, CC398/CC22, CC22/CC8 and SK41/SK42 of S. aureus. Table S7B: Proteins with a higher and lower abundance in CC398 versus CC8 exoproteomes. Table S7C: Proteins with a higher and lower abundance in CC398 versus CC22 exoproteomes. Table S7D: Proteins with a higher and lower abundance in CC22 versus CC8 exoproteomes. Table S7E: Proteins with a higher and lower abundance in CC398 versus CC8 and CC22 exoproteomes.
- Table S8 - The 99 percentile normalized LFQ intensity values of 869 secreted proteins in the secretome of the eight S. aureus isolates of CC8, CC22 and CC398.
