# Supplementary material for: Comparative Secretome Analyses of Human and Zoonotic Staphylococcus aureus Isolates CC8, CC22, and CC398
Source: Mol Cell Proteomics. 2018 Sep 10;17(12):2412–33. doi: 10.1074/mcp.RA118.001036 (PMC6283302; doi:10.1074/mcp.RA118.001036)

MS/MS Spectra of proteins with only 1 identified unique peptide in all strains and 2/3 of replicates

MS-Viewer key r1mmcmhlpi

PanID/ RKI-no.: SAUPAN003402000_1

Protein_Ids: 27969_02259;18787_01443

(Allelic variant: SAUPAN003402000_2 - 38876_00169)

Raw file	Scan number	Scan index	Sequence
42_1_C	10323	6899	AVNLVSFEYNVK

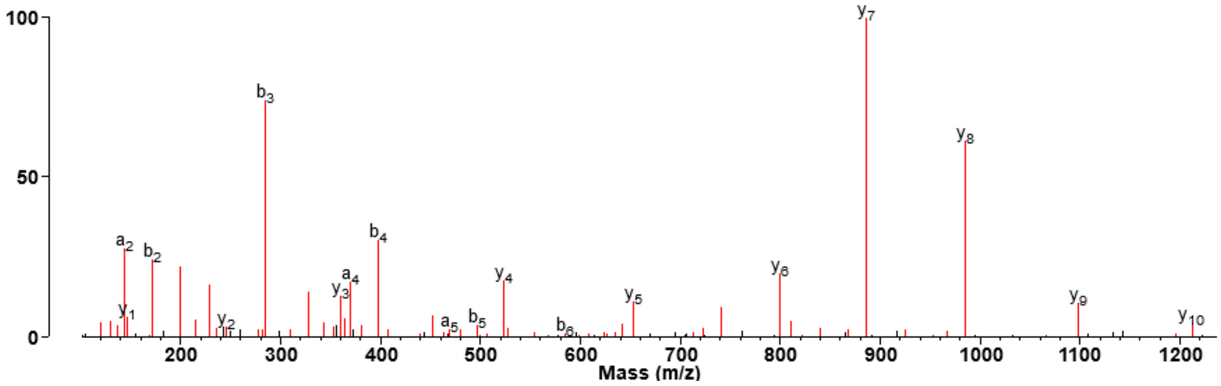

-

PanID/ RKI-no.: SAUPAN005012000_1

Protein_Ids: 38888_02126;18787_01769

(Allelic variant: SAUPAN005012000_1 - 18787_01825)

Raw file	Scan number	Scan index	Sequence
885_2B	1871	146	KDDTDLK

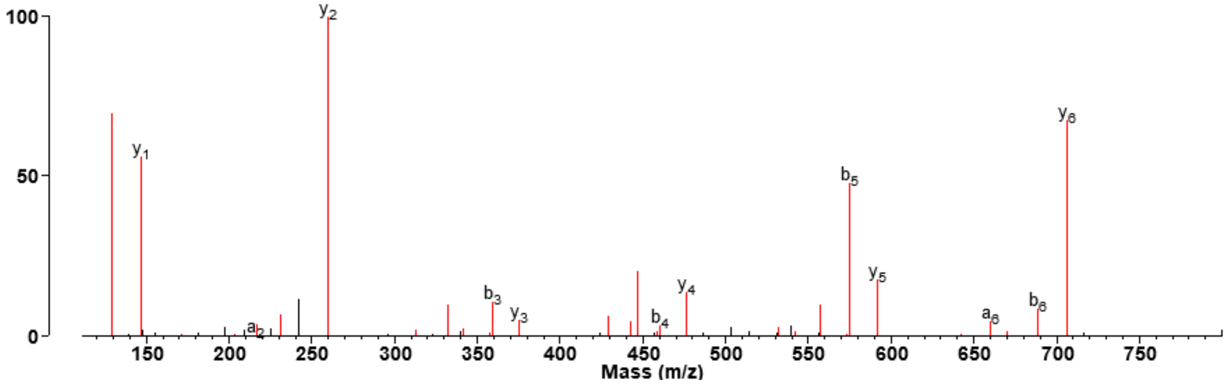

-

PanID/ RKI-no.: SAUPAN005914000

Protein_Ids: 27969_01416

(Allelic variant: none)

Raw file	Scan number	Scan index	Sequence
42_2_C	5565	2964	IKPSNDTTSSTSIPIS

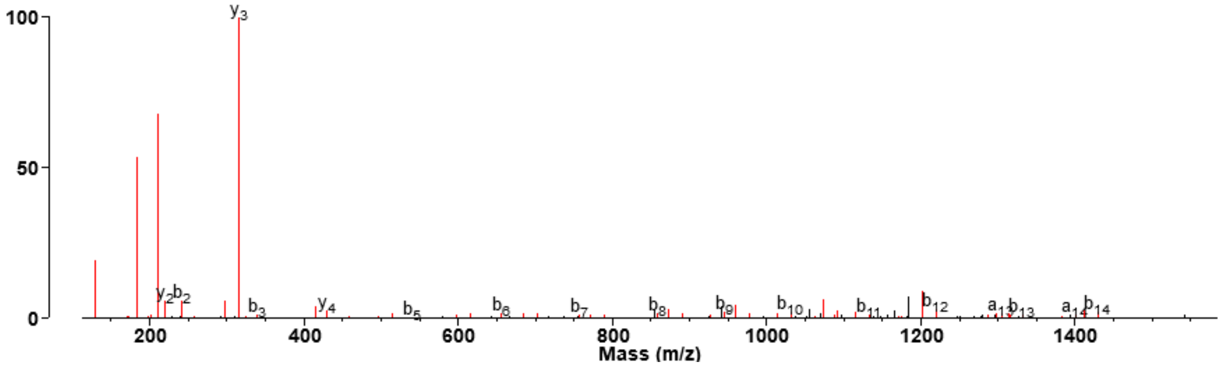

PanID/ RKI-no.: SAUPAN005914000
Protein_Ids: 27969_01416
(Allelic variant: none)

Raw file	Scan number	Scan index	Sequence
41_2_C	7750	5025	NFESLIR

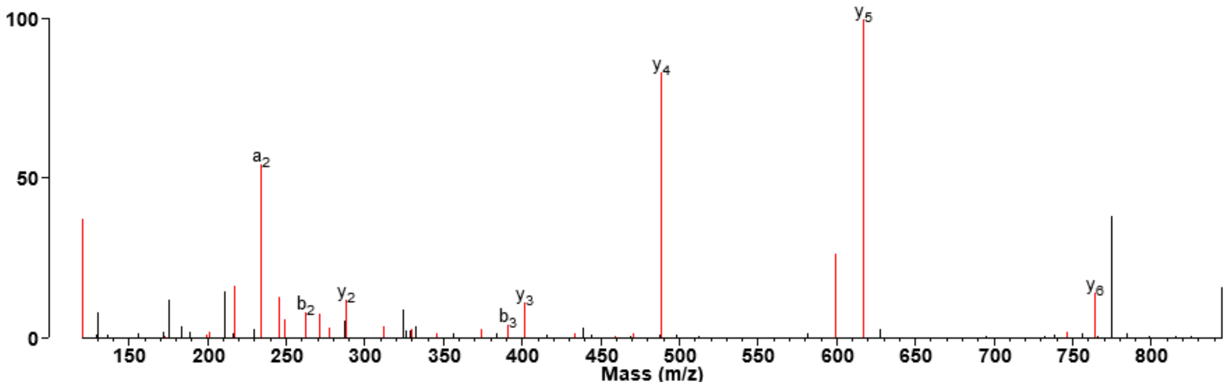

-

PanID/ RKI-no.: SAUPAN004211000

Protein_Ids: 18787_00544

(Allelic variant: none)

Raw file	Scan number	Scan index	Sequence
42_2_C	20242	15375	LVPIETDEEWDMIEEVVNTEMEE

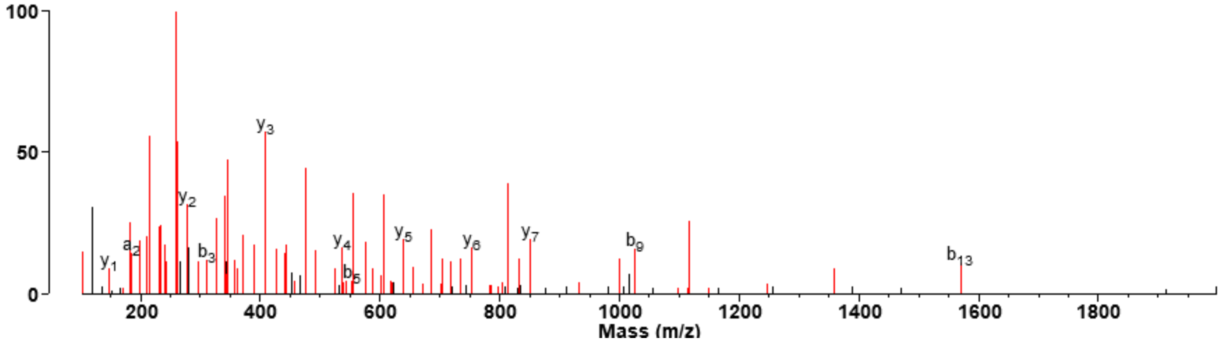

PanID/ RKI-no.: SAUPAN004211000
Protein_Ids: 18787_00544
(Allelic variant: none)

Raw file	Scan number	Scan index	Sequence
41_3_C	20248	15359	LVPIETDEEWDMIEEVVNTEMEE

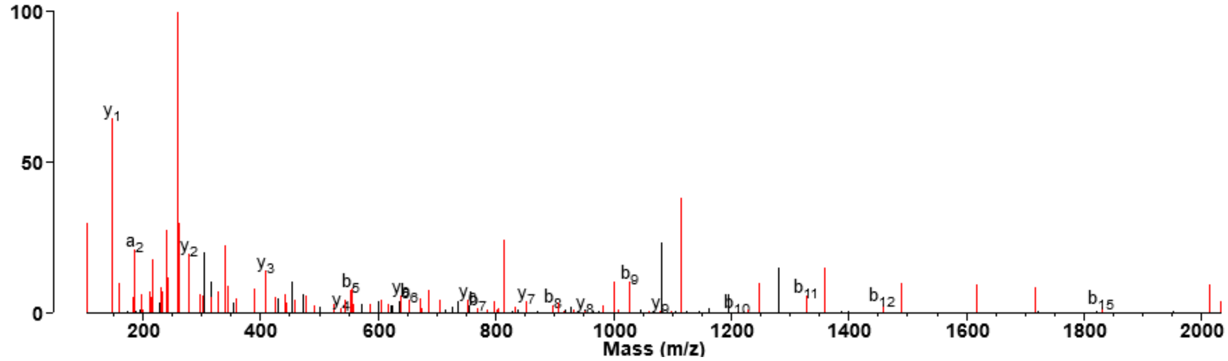

PanID/ RKI-no.: SAUPAN004211000
Protein_Ids: 18787_00544
(Allelic variant: none)

Raw file	Scan number	Scan index	Sequence
42_1_C	18463	13786	LVPIETDEEWDMIEEVVNTEMEE

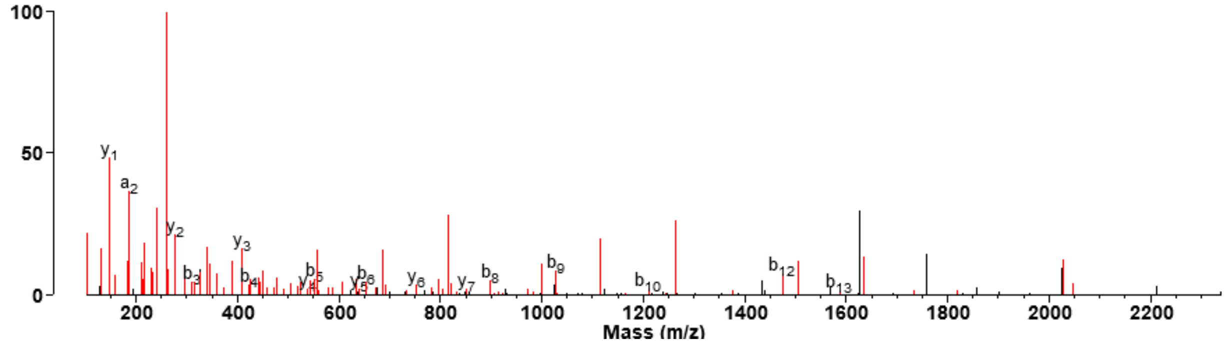

PanID/ RKI-no.: SAUPAN004211000
Protein_Ids: 18787_00544
(Allelic variant: none)

Raw file	Scan number	Scan index	Sequence
41_2_C	16207	12139	LVPIETDEEWDMIEEVVNTEMEE

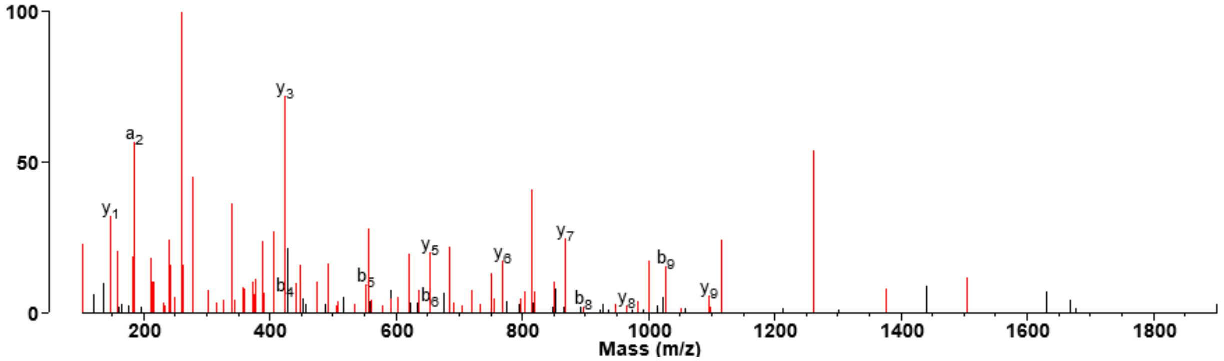

PanID/ RKI-no.: SAUPAN004211000
Protein_Ids: 18787_00544
(Allelic variant: none)

Raw file	Scan number	Scan index	Sequence
42_3_C	18399	13807	LVPIETDEEWDMIEEVVNTEMEE

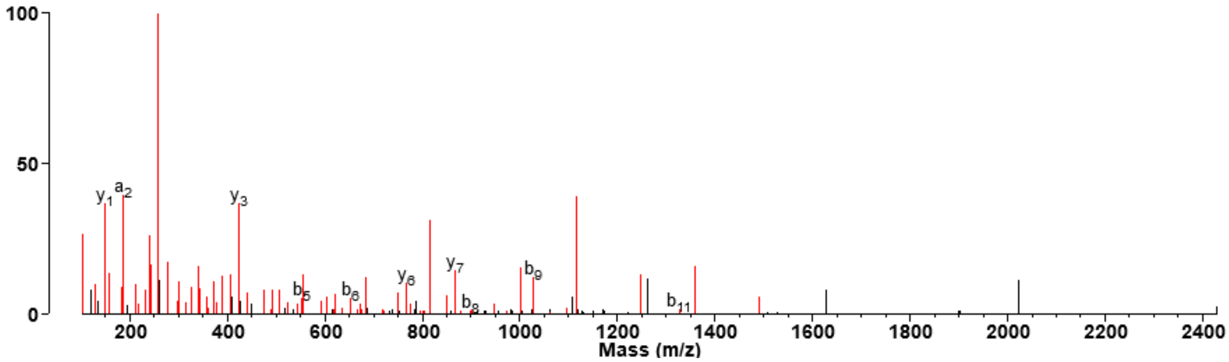

PanID/ RKI-no.: SAUPAN004211000
Protein_Ids: 18787_00544
(Allelic variant: none)

Raw file	Scan number	Scan index	Sequence
41_1_C	12552	9119	VLEFYHPEFK

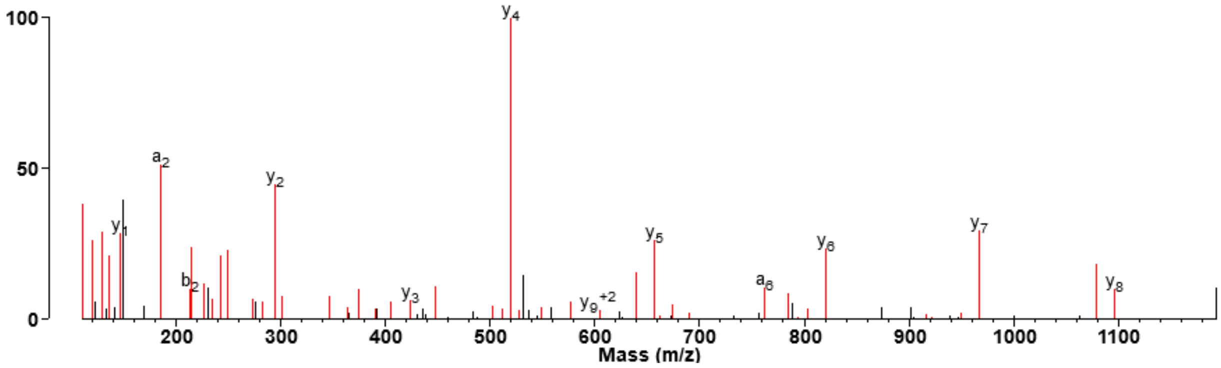

-

PanID/ RKI-no.: SAUPAN002102000_1

Protein_Ids: 18787_02434

(Allelic variant: with only 1 unique peptide SAUPAN002102000_2)

Raw file	Scan number	Scan index	Sequence
749_4C	23132	20052	ELAAILSEASGTEIKYEPVSLETFAK

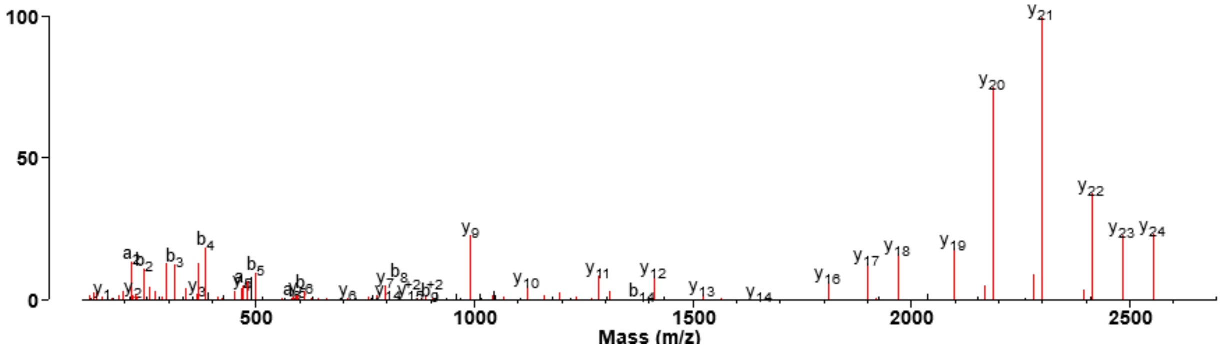

PanID/ RKI-no.: SAUPAN002102000_1
Protein_Ids: 18787_02434
(Allelic variant: with only 1 unique peptide SAUPAN002102000_2)

Raw file	Scan number	Scan index	Sequence
787_1B	22281	19408	GMDTVVFIPSIIHPSFK

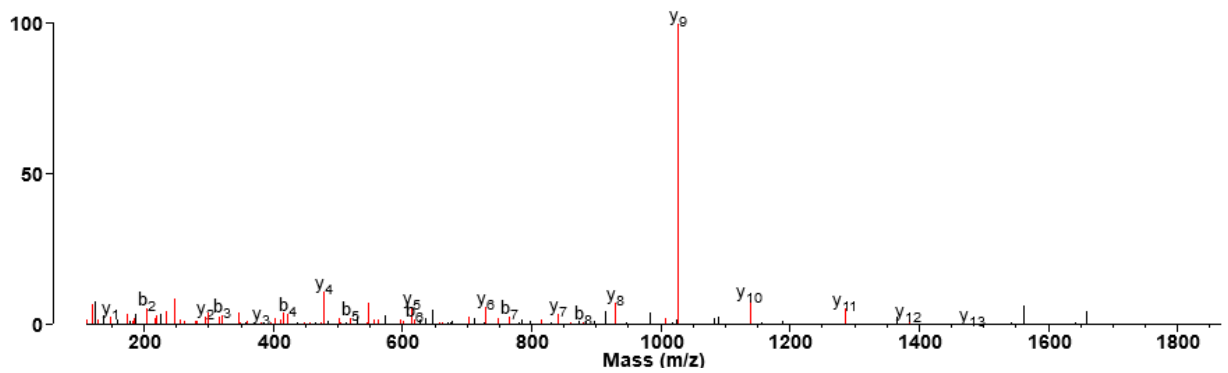

PanID/ RKI-no.: SAUPAN002102000_1
Protein_Ids: 18787_02434
(Allelic variant: with only 1 unique peptide SAUPAN002102000_2)

Raw file	Scan number	Scan index	Sequence
787_1B	15219	12924	YEPVSLETFAK

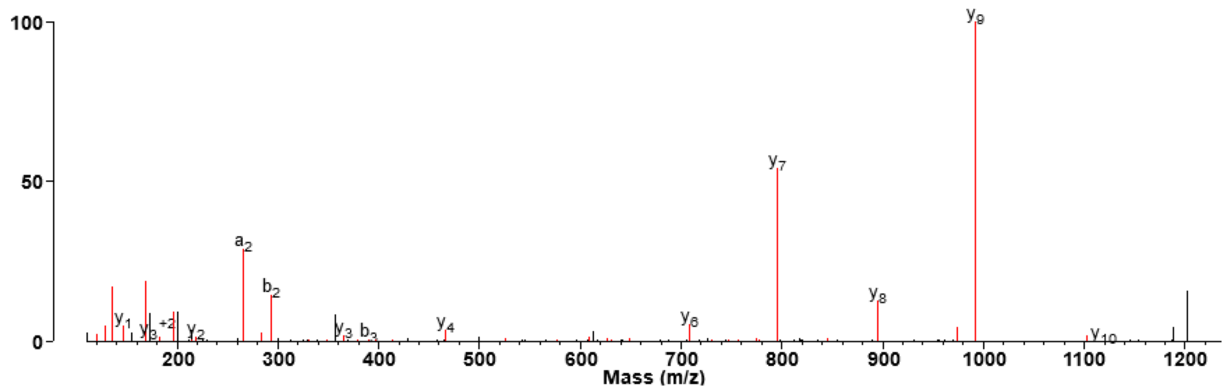

-

PanID/ RKI-no.: SAUPAN002102000_2

Protein_Ids: 27969_00555

(Allelic variant: with only 1 unique peptide SAUPAN002102000_1)

Raw file	Scan number	Scan index	Sequence
885_1C	21694	18179	GIDTVVFIPSIIHPSFK

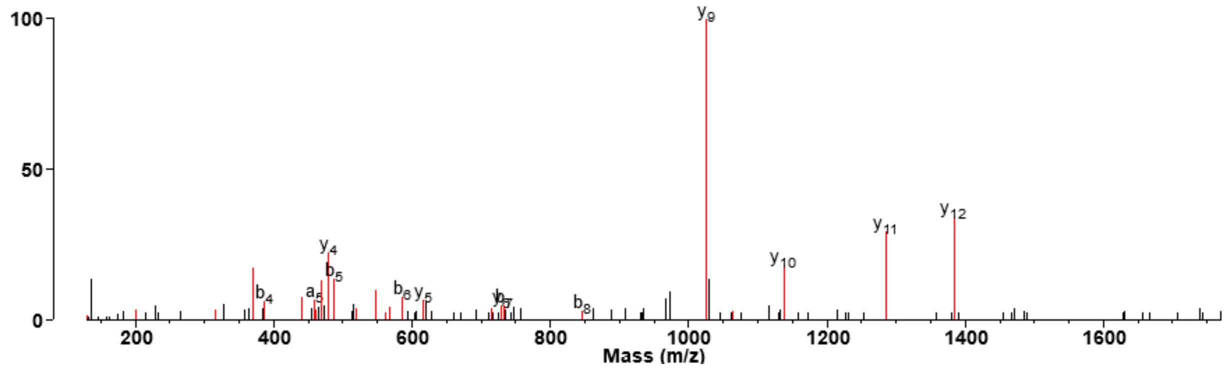

PanID/ RKI-no.: SAUPAN002102000_2
Protein_Ids: 27969_00555
(Allelic variant: with only 1 unique peptide SAUPAN002102000_1)

Raw file	Scan number	Scan index	Sequence
885_3B	23913	20913	GIDTVVFIPSIIHPSFK

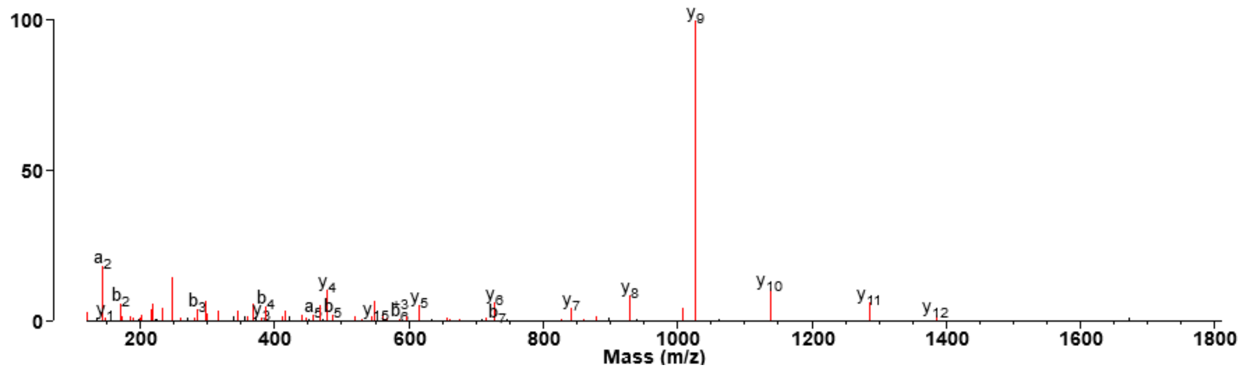

PanID/ RKI-no.: SAUPAN002113000_1
Protein_Ids: 18787_02429
(Allelic variant: SAUPAN002113000_2- 27969_00561; SAUPAN002113000_3 - 38876_00555)

Raw file	Scan number	Scan index	Sequence
749_2B	18803	16548	VDGGDLHASIDSFLIQK

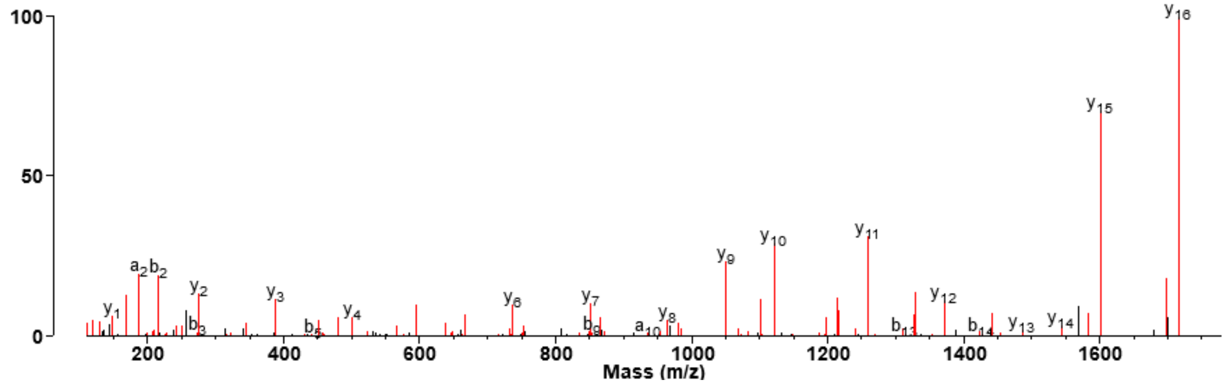

-

PanID/ RKI-no.: SAUPAN003398000_1

Protein_Ids: 18787_01587

(Allelic variant: SAUPAN003398000_2 - 38876_00167)

Raw file	Scan number	Scan index	Sequence
749_1c	2692	979	GNTIAVVR

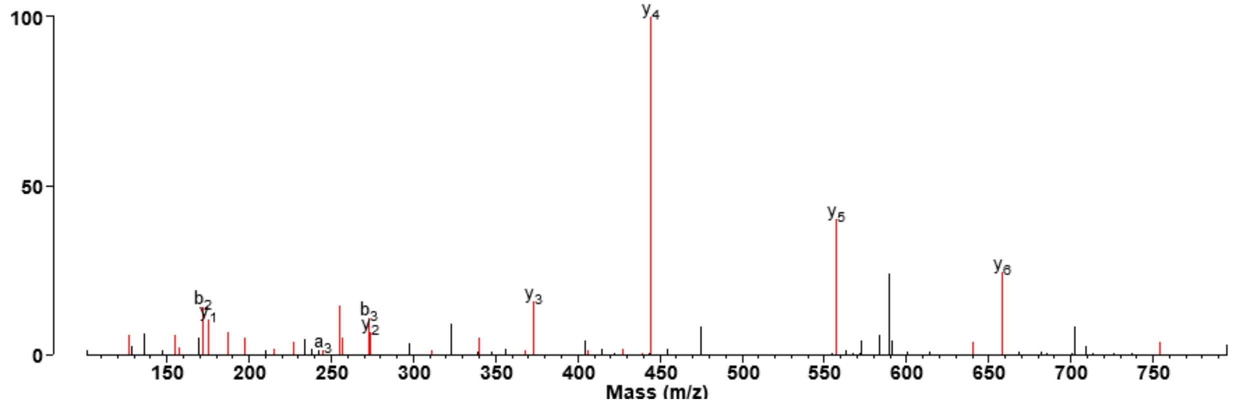

PanID/ RKI-no.: SAUPAN003398000_1
Protein_Ids: 18787_01587
(Allelic variant: SAUPAN003398000_2 - 38876_00167)

Raw file	Scan number	Scan index	Sequence
749_4C	14266	11899	TVEDLYVIAK

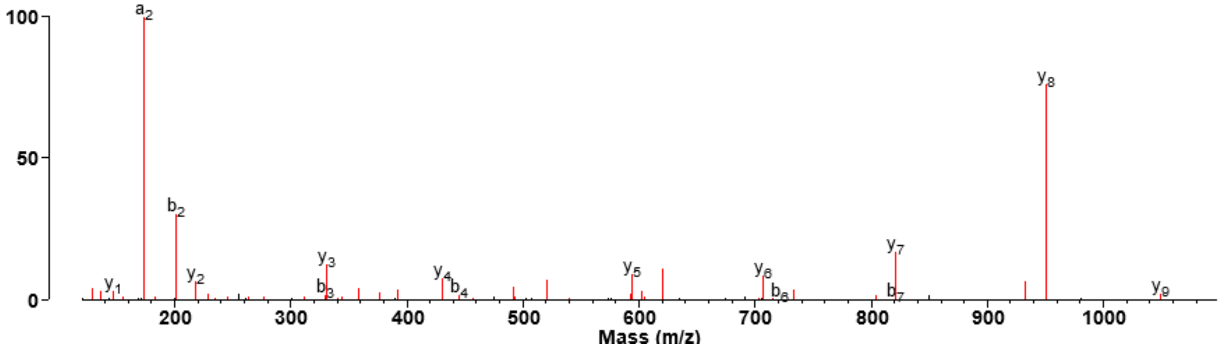

-

PanID/ RKI-no.: SAUPAN001817000

Protein_Ids: 24749_00673

(Allelic variant: none)

Raw file	Scan number	Scan index	Sequence
749_2C	12116	9328	IGEAGIIQNSIVQK

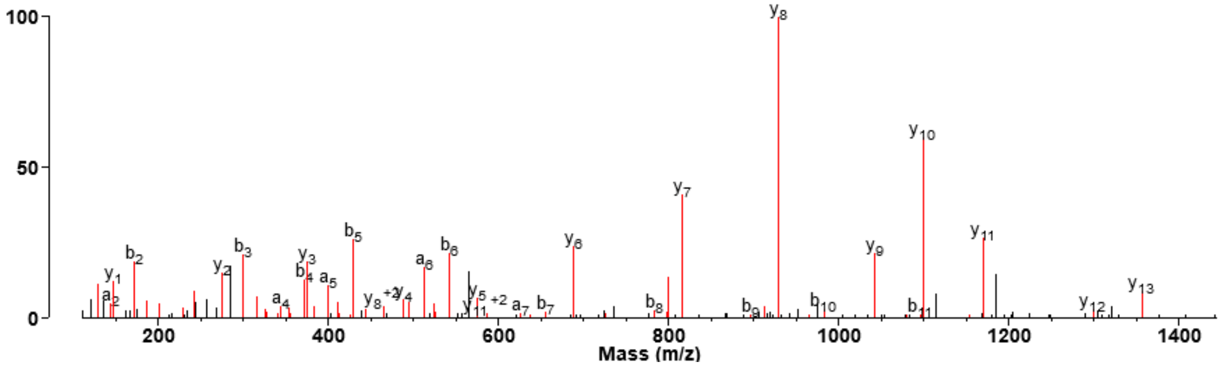

PanID/ RKI-no.: SAUPAN003392000_1

Protein_Ids: 18787_01589;38876_00165

(Allelic variant: with only 1 unique peptide SAUPAN003392000_2)

Raw file	Scan number	Scan index	Sequence
749_4C	3338	1849	HHETISHTTF

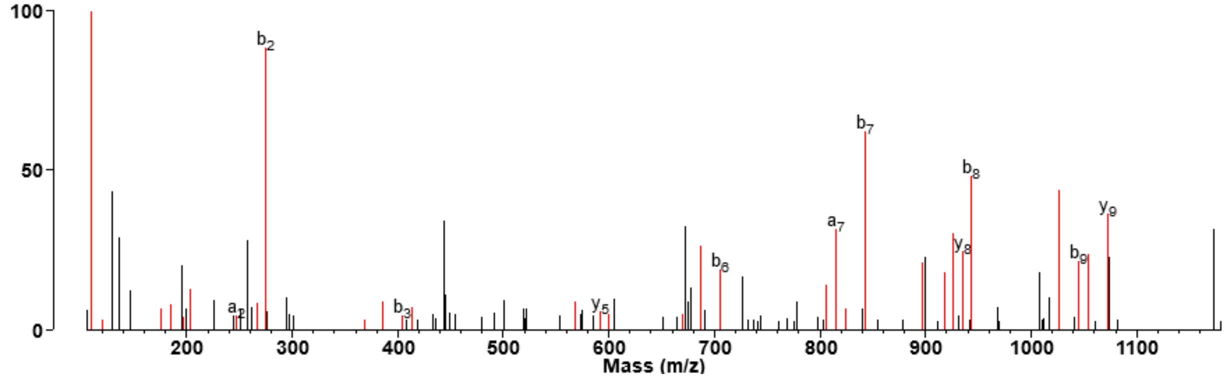

PanID/ RKI-no.: SAUPAN003392000_1
Protein_Ids: 18787_01589;38876_00165
(Allelic variant: with only 1 unique peptide SAUPAN003392000_2)

Raw file	Scan number	Scan index	Sequence
749_4C	3300	1815	HHETISHTTF

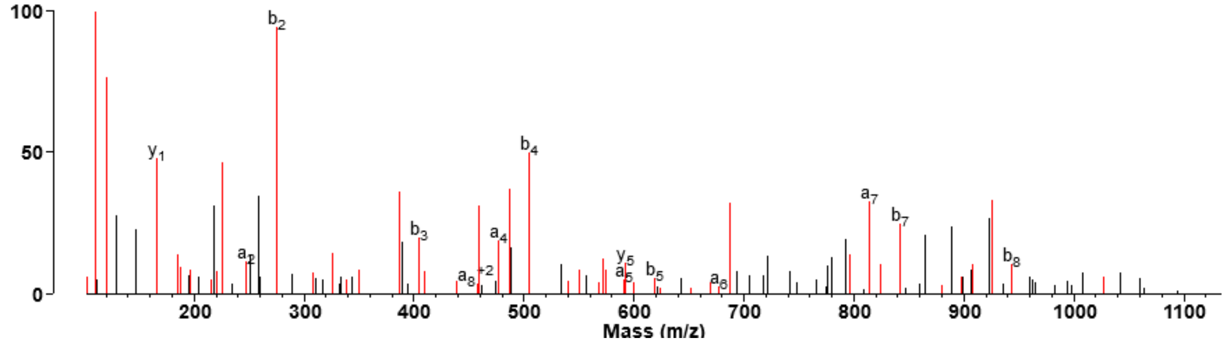

-

PanID/ RKI-no.: SAUPAN003392000_2

Protein_Ids: 27969_02263

(Allelic variant: with only 1 unique peptide SAUPAN003392000_1)

Raw file	Scan number	Scan index	Sequence
885_1C	4568	2928	QHETISHTTF

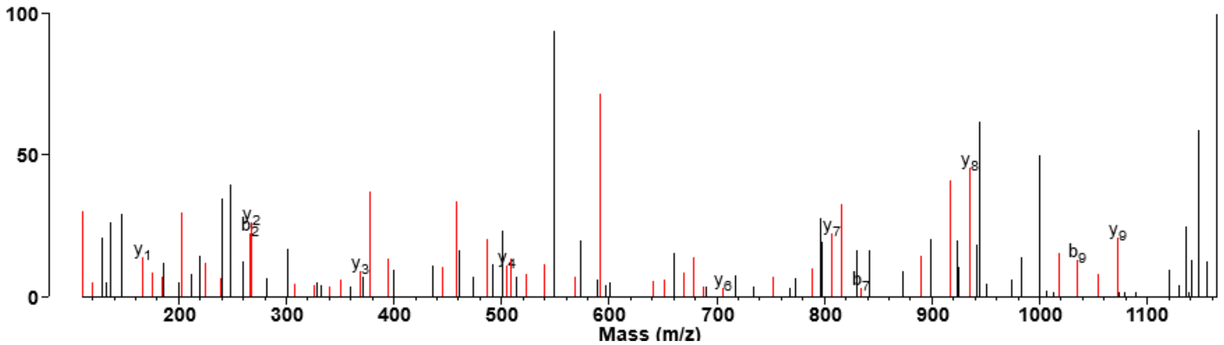

PanID/ RKI-no.: SAUPAN003392000_2
Protein_Ids: 27969_02263
(Allelic variant: with only 1 unique peptide SAUPAN003392000_1)

Raw file	Scan number	Scan index	Sequence
885_1C	21066	17632	SSMEESYAEVLIDDQTLQGTVNFK

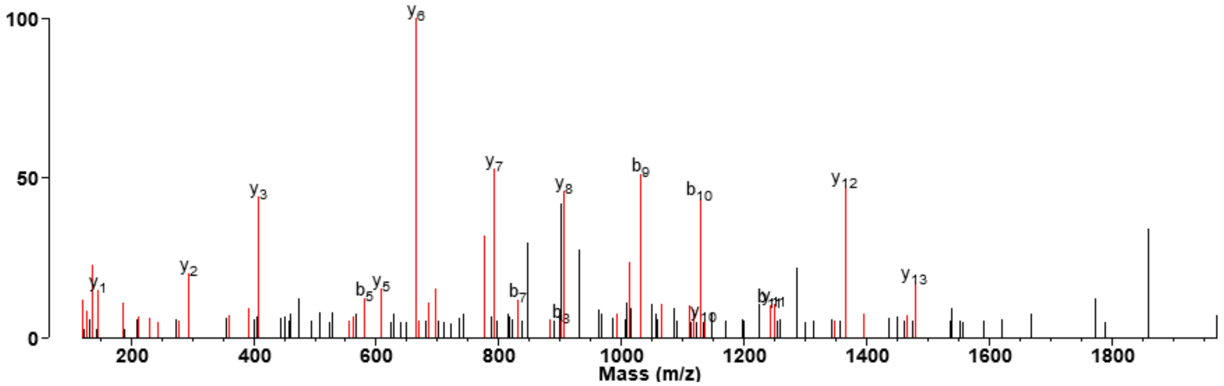

-

PanID/ RKI-no.: SAUPAN003978000

Protein_Ids: 24749_00642

(Allelic variant: none)

Raw file	Scan number	Scan index	Sequence
749_1c	3914	1971	APLKNGHEDLAK

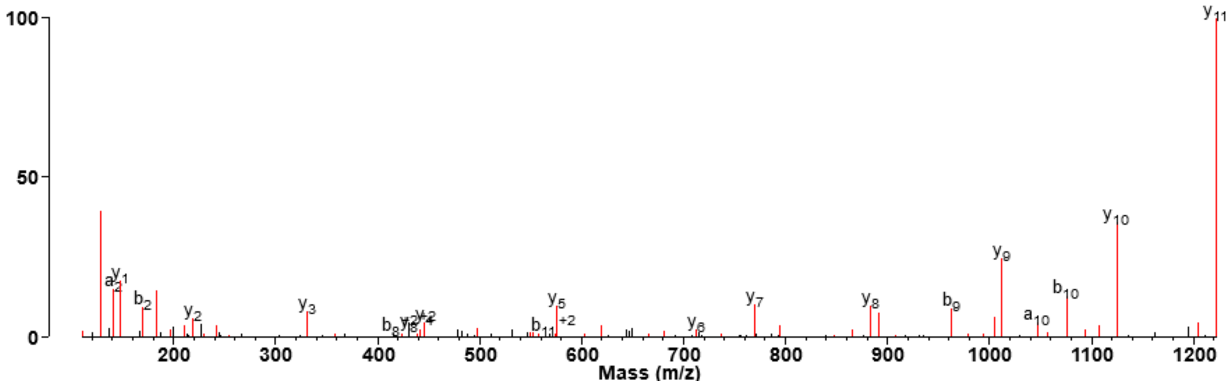

PanID/ RKI-no.: SAUPAN003978000
Protein_Ids: 24749_00642
(Allelic variant: none)

Raw file	Scan number	Scan index	Sequence
749_1c	23516	17503	FYVDRVFDLWEG

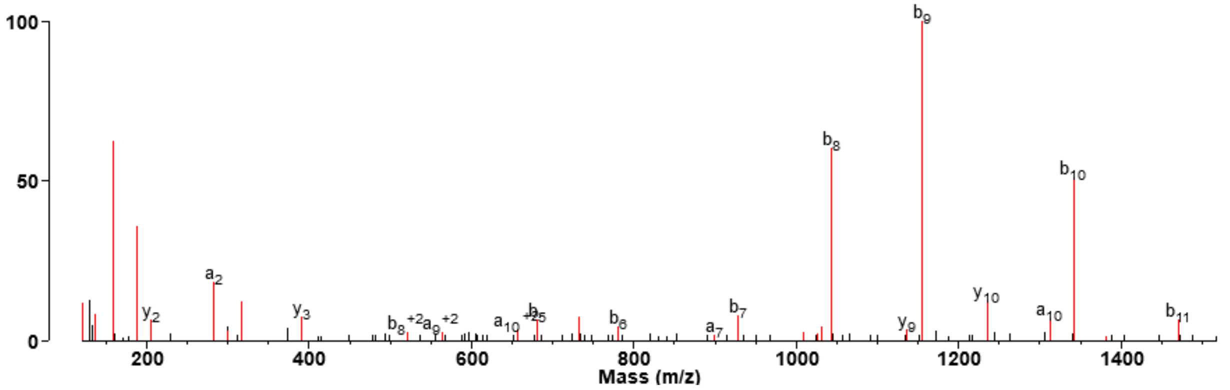

PanID/ RKI-no.: SAUPAN003978000
Protein_Ids: 24749_00642
(Allelic variant: none)

Raw file	Scan number	Scan index	Sequence
749_4C	2967	1522	KVDMNETQDNVK

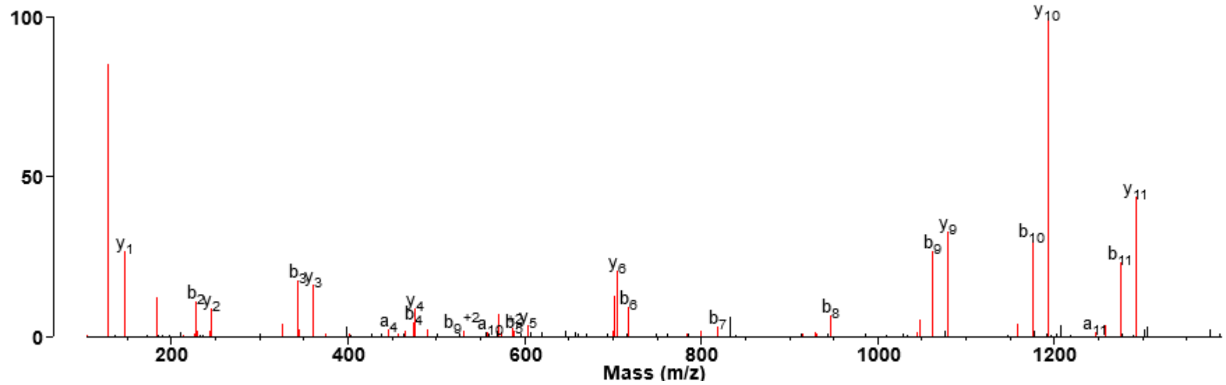

PanID/ RKI-no.: SAUPAN003978000
Protein_Ids: 24749_00642
(Allelic variant: none)

Raw file	Scan number	Scan index	Sequence
749_4C	2966	1521	KVDMNETQDNVK

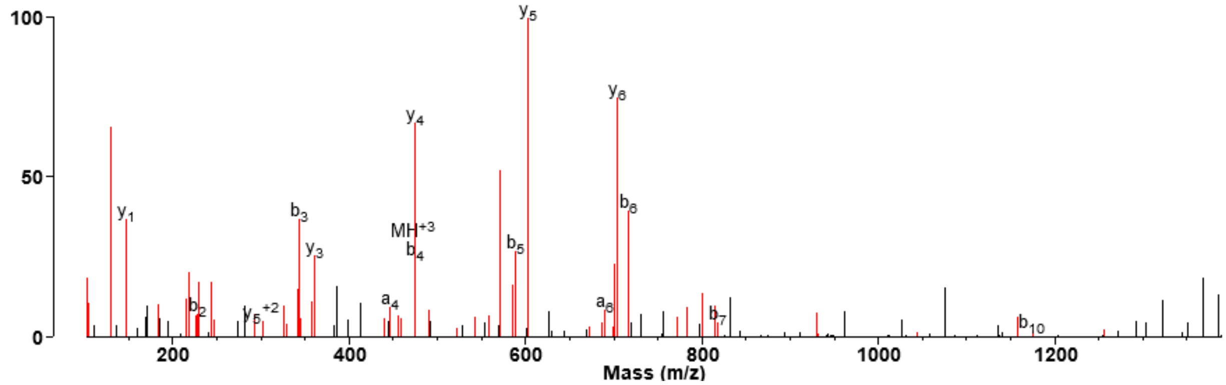

Supplement: supplemental Table S6 [file 140073_0_supp_183725_pd9z4x.pdf]
