# Supplementary material for: Comparative Secretome Analyses of Human and Zoonotic Staphylococcus aureus Isolates CC8, CC22, and CC398
Source: Mol Cell Proteomics. 2018 Sep 10;17(12):2412–33. doi: 10.1074/mcp.RA118.001036 (PMC6283302; doi:10.1074/mcp.RA118.001036)
Supplement: supplemental Table S6 [file 140073_0_supp_183733_pdkz4y.pdf]

Figure S2

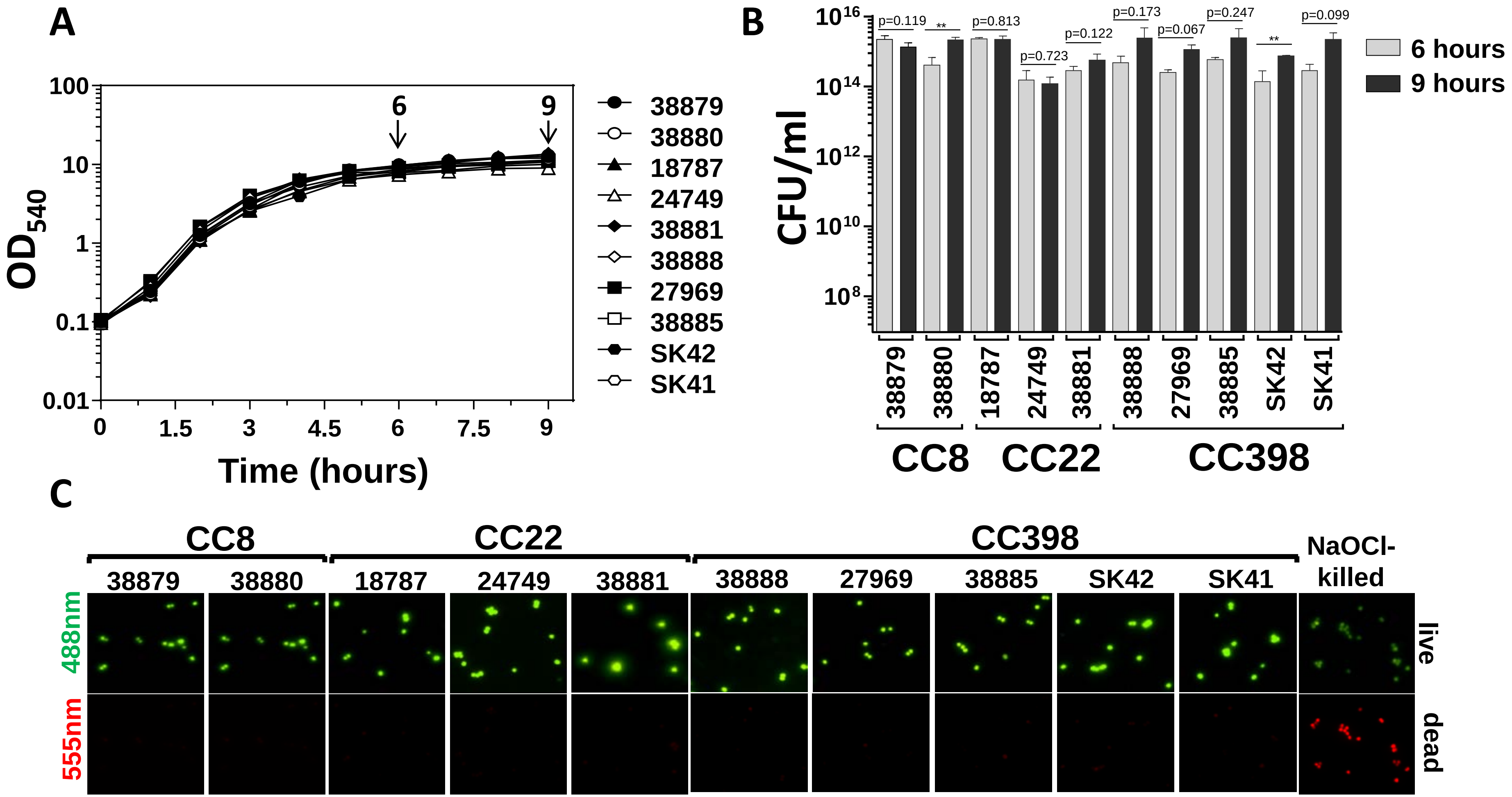

Figure S2: Growth curves (A), survival counts (B) and live-dead assay (C) of *S. aureus* strains of CC8, CC22 and CC398 during the stationary phase. *S. aureus* strains were grown in TSB medium and cell lysis was analyzed after 6 and 9 hours growth during the stationary phase by CFU counting **(B)** and after 9 hours growth with live-dead assay using BacLight™ bacterial viability kit **(C)**. No significant differences were measured in cell lysis during the stationary phase. As control was used the 9 hour culture killed with 1 mM NaOCl.
