# Supplementary material for: Comparative Secretome Analyses of Human and Zoonotic Staphylococcus aureus Isolates CC8, CC22, and CC398
Source: Mol Cell Proteomics. 2018 Sep 10;17(12):2412–33. doi: 10.1074/mcp.RA118.001036 (PMC6283302; doi:10.1074/mcp.RA118.001036)
Supplement: supplemental Table S6 [file 140073_0_supp_183734_pdyz4y.pdf]

Figure S3

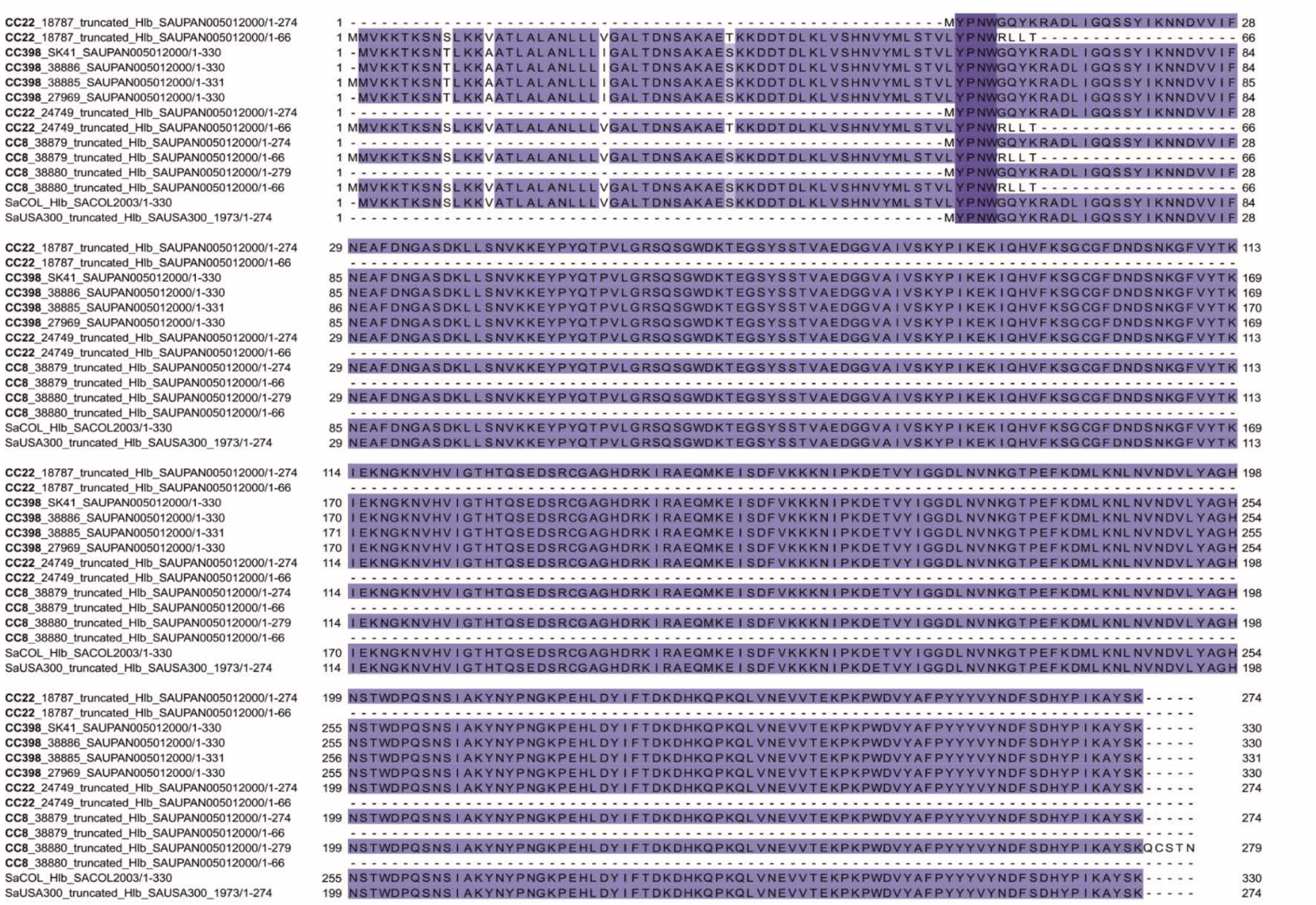

Figure S3: Alignment of ß-hemolysin protein variants as revealed by the FASTA protein sequences that are translated from the genome sequences of the selected *S. aureus* isolates from CC8, CC22 and CC398. Amino acid sequences were aligned using ClustalΩ and presented using Jalview. Intensity of the blue color is based on sequence identities. The selected four CC398 isolates all encode full-length Hlb protein of 330-331 aa. In contrast, the two CC8 and two CC22 isolates encode only 66 aa N-terminal and 274 aa C-terminal variants of truncated Hlb proteins that are non-functionally.
